# Tankyrase inhibition ameliorates lipid disorder via suppression of PGC-1α PARylation in *db/db* mice

**DOI:** 10.1038/s41366-020-0573-z

**Published:** 2020-04-21

**Authors:** Hong Wang, Sara Kuusela, Rita Rinnankoski-Tuikka, Vincent Dumont, Rim Bouslama, Usama Abo Ramadan, Jo Waaler, Anni-Maija Linden, Nai-Wen Chi, Stefan Krauss, Eija Pirinen, Sanna Lehtonen

**Affiliations:** 1 Department of Pathology, University of Helsinki, Helsinki, Finland; 2 Research Program for Clinical and Molecular Metabolism, Faculty of Medicine, University of Helsinki, Helsinki, Finland; 3 Experimental MRI Laboratory, University of Helsinki and Helsinki University Hospital, Helsinki, Finland; 4 Department of Immunology and Transfusion Medicine, Oslo University Hospital, Oslo, Norway; 5 Hybrid Technology Hub—Centre of Excellence, Institute of Basic Medical Sciences, University of Oslo, Oslo, Norway; 6 Department of Pharmacology, University of Helsinki, Helsinki, Finland; 7 Endocrine Service, VA San Diego Healthcare System, San Diego, CA USA; 8 Present Address: Minerva Institute for Medical Research, Helsinki, Finland

**Keywords:** Diabetes, Mouse

## Abstract

**Objective:**

Human TNKS, encoding tankyrase 1 (TNKS1), localizes to a susceptibility locus for obesity and type 2 diabetes mellitus (T2DM). Here, we addressed the therapeutic potential of G007-LK, a TNKS-specific inhibitor, for obesity and T2DM.

**Methods:**

We administered G007-LK to diabetic *db/db* mice and measured the impact on body weight, abdominal adiposity, and serum metabolites. Muscle, liver, and white adipose tissues were analyzed by quantitative RT-PCR and western blotting to determine TNKS inhibition, lipolysis, beiging, adiponectin level, mitochondrial oxidative metabolism and mass, and gluconeogenesis. Protein interaction and PARylation analyses were carried out by immunoprecipitation, pull-down and in situ proximity ligation assays.

**Results:**

TNKS inhibition reduced body weight gain, abdominal fat content, serum cholesterol levels, steatosis, and proteins associated with lipolysis in diabetic *db/db* mice. We discovered that TNKS associates with PGC-1α and that TNKS inhibition attenuates PARylation of PGC-1α, contributing to increased PGC-1α level in WAT and muscle in *db/db* mice. PGC-1α upregulation apparently modulated transcriptional reprogramming to increase mitochondrial mass and fatty acid oxidative metabolism in muscle, beiging of WAT, and raised circulating adiponectin level in *db/db* mice. This was in sharp contrast to the liver, where TNKS inhibition in *db/db* mice had no effect on PGC-1α expression, lipid metabolism, or gluconeogenesis.

**Conclusion:**

Our study unravels a novel molecular mechanism whereby pharmacological inhibition of TNKS in obesity and diabetes enhances oxidative metabolism and ameliorates lipid disorder. This happens via tissue-specific PGC-1α-driven transcriptional reprogramming in muscle and WAT, without affecting liver. This highlights inhibition of TNKS as a potential pharmacotherapy for obesity and T2DM.

## Introduction

Adipose tissue and skeletal muscle are important regulators of the metabolic homeostasis of the whole body. White adipose tissue (WAT) produces a number of factors that regulate energy metabolism, and, consequently, WAT dysfunction plays a critical role in the pathogenesis of obesity [1]. The physiological effects of obesity are reflected on nearly all the tissues through adverse metabolic repercussions, such as dyslipidemia, insulin resistance, and hepatic steatosis. Muscle is highly active in lipid oxidation and glucose clearance. In people with type 2 diabetes mellitus (T2DM), the rate of lipid oxidation in muscle is reduced [2]. Studies have revealed lowered functional capacity of mitochondria in muscle in T2DM [3], associating mitochondrial dysfunction with insulin resistance, but their causal relationship remains unclear [4].

Peroxisome proliferator-activated receptor γ (PPARγ) coactivator 1α (PGC-1α) is a central regulator of mitochondrial and energy metabolism [5]. It is regulated by complex tissue-specific processes that include regulation of gene expression, posttranslational modification, and protein stabilization [6]. PGC-1α co-activates transcription factors that boost mitochondrial biogenesis and oxidative metabolism in muscle and adipose tissue and promote gluconeogenesis in liver [7–9]. PGC-1α expression is repressed in muscle and adipose tissue in patients with obesity and T2DM [10, 11], and increased in liver in rodent models of diabetes [12]. Given these divergent effects of PGC-1α between endocrine organs, an ideal PGC-1α-directed therapeutic agent should boost its activity in muscle and WAT but not in liver. As a drug target PGC-1α itself is challenging, and an appealing approach might be to target a protein that regulates its activity or stability in a tissue-specific manner.

Tankyrases (TNKSs) are members of the poly (ADP-ribose) polymerase (PARP) family, which catalyze posttranslational modification (PARylation) of proteins by transferring the ADP-ribose moiety of NAD^+^ to target proteins [13]. The PARylation activity of TNKSs affects the functions and stability of target proteins and thereby modulates diverse cellular processes including Wnt signaling, vesicular trafficking, maintenance of telomeres, osteoblast differentiation, and energy metabolism [13–18]. TNKS-mediated PARylation depends on the sequence of the TNKS-binding motif and the structural features of the binding partner [19]. Consequently, not all binding partners become PARylated [19], and some even inhibit the PARylation activity of TNKSs [20]. In humans, *TNKS* (the gene encoding tankyrase 1, TNKS1) localizes to chromosome 8p23.1, a susceptibility locus for T2DM [21], and variants of *TNKS* associate with early-onset obesity [22]. These findings suggest a role for TNKS1 in metabolic disorders. In line with this, depletion of TNKS1 in mice enhances energy expenditure, fatty acid oxidation (FAO) and insulin sensitivity, and reduces adiposity [17]. Furthermore, adipocyte-specific disruption of the PARylation activity of TNKS1 enhances glucose tolerance and insulin sensitivity in female mice [18]. Suppression of TNKS1 and the closely related TNKS2 with a TNKS-specific inhibitor in high fat diet-fed mice lowers fasting glucose and improves insulin sensitivity [18]. However, whether long-term pharmacological inhibition of TNKSs improves lipid metabolism and serves as a therapeutic tool to treat obesity and T2DM remain uncharacterized. To fill this gap and define the underlying molecular mechanisms, this study used *db/db* mice as a diabetes model to investigate the effect of pharmacological TNKS inhibition, focusing on PGC-1α-regulated oxidative metabolism in WAT and skeletal muscle.

## Materials and methods

### Animal experiments

Animal experiments were performed in compliance with the national ethical guidelines set by the European Union and were approved by the National Animal Experiment Board. Diabetic male C57BLKS/J *db/db* and nondiabetic *db/+* mice (Taconic Biosciences, Ry, Denmark) were individually housed with access to chow and water ad libitum. *db/db* and *db/+* mice were randomly divided into groups (*n* = 10 per group) and fed from 6 weeks of age for 15 weeks with Purina 5001 (Research Diets, Inc., New Brunswick, NJ, USA) or the same chow containing G007-LK 100 mg/kg (Research Diets, Inc.). This provided a G007-LK average dosing of 14 mg/kg/d for *db/db* and 12 mg/kg/d for *db/+* mice. At the end of the experiment, mice were euthanized, and blood was collected by cardiac puncture. Freshly dissected epididymal WAT, quadriceps muscle, and liver tissue were snap-frozen in liquid nitrogen, embedded in Tissue-Tek^®^ OCT compound (Sakura, Alphen aan den Rijn, The Netherlands), or fixed in 10% formalin followed by embedding in paraffin.

### Magnetic resonance imaging (MRI)

MRI was performed under anesthesia after 10 weeks of treatment with a 4.7-T scanner (PharmaScan; Bruker BioSpin, Ettlingen, Germany) and a series of T2-weight axial slices were analyzed to determine abdominal fat composition with the Medical Image Processing, Analysis, and Visualization software (MIPAV, v.7.0.1 NIH, Bethesda, MD, USA).

### Metabolite measurements

For serum lipid profile, blood samples collected by cardiac puncture were analyzed with a Siemens ADVIA 1650 analyzer (Diamond Diagnostics, Holliston, MA, USA) at the Biochemical Analysis Core for Experimental Research, University of Helsinki. Serum glycerol was measured with a commercial Kit (Sigma-Aldrich, St. Louis, MO, USA).

### Histology

Hepatic lipid accumulation was analyzed by Oil Red O staining of frozen liver sections. Images were captured with an Eclipse 800 microscope (Nikon Inc., Melville, NY, USA) equipped with a digital camera (Spot Imaging Solutions, Sterling Heights, MI, USA). Lipid content was quantified with Image-Pro Analyzer 6.0.

### qRT-PCR

qRT-PCR was performed as described [23] at least three times with three technical replicates for each sample. The median Ct values were used for analysis with the 18S ribosomal RNA as an internal control. The primer pairs are listed in Table S1.

### Western blotting

Tissues were lysed in chilled RIPA buffer using Dounce homogenizer, and western blotting was performed at least three times as previously described [23]. The antibodies used are listed in Table S2.

### Protein–protein interaction and PARylation assays

Immunoprecipitation was performed with muscle lysate of normal ICR mice as described [24]. The antibodies used are listed in Table S2.

Duolink in situ proximity ligation assay (PLA) was carried out according to the manufacturer’s instructions (Sigma-Aldrich, St. Louis, MO, USA). Briefly, antigen retrieval of deparaffinized 4 µm paraffin sections was carried out with 10 mM citrate buffer, pH 6. Frozen sections of WAT (25 µm) were fixed with 2% PFA. Muscle and WAT sections were permeabilized with 0.1% or 0.3% Triton-X100 for 30 or 15 min at RT, respectively. Sections were blocked with CAS-block (Invitrogen) for 1 h at RT and incubated O/N at +4 °C with primary antibodies diluted in ChemMate™ (DakoCytomation, Glostrup, Denmark). Primary antibodies used are listed in Table S2. Images were captured using Zeiss Axioplan2 microscope (Carl Zeiss Microscopy, Thornwood, NY, USA) and quantified with the Duolink Image Tool software (Olink Bioscience, Uppsala, Sweden).

Pull-down assay with WWE Affinity Resin Kit (Tulip Biolabs, West Point, PA, USA) was performed according to the manufacturer’s instruction. PARylated PGC-1α and TNKS1 were detected by immunoblotting the affinity precipitates with anti-PGC-1α (EMD Millipore) and anti-TNKS1/2 (Santa Cruz Biotechnology) IgGs.

### Cell culture, fatty acid oxidation (FAO), citrate synthase activity, and mitochondrial respiration

Murine C2C12 myoblasts (ATCC, Manassas, VA, USA) were maintained in high glucose (4500 mg/l) Dulbecco’s modified Eagle’s medium (DMEM) (Sigma-Aldrich, St. Louis, MO, USA) supplemented with 10% fetal bovine serum (FBS), 1% nonessential amino acids (Thermo Fisher Scientific, Waltham, MA, USA), 2% Hepes, and 1% penicillin–streptomycin. Myoblasts were differentiated into myotubes in DMEM supplemented with 2% horse serum instead of 10% FBS. On differentiation day 4, the cells were treated with G007-LK or vehicle (DMSO) (Sigma-Aldrich) for 24 h in low glucose (1000 mg/l) DMEM differentiation medium supplemented with 1% albumin-bound oleic acid (Sigma-Aldrich).

Oleic acid oxidation in differentiated C2C12 cells was measured as previously described [25] with minor modifications. Briefly, the β-oxidation was carried out at 37 °C for 2 h in medium containing Krebs–Henseleit buffer, 3.3% albumin-bound oleic acid, 25 µM L-carnitine (Sigma-Aldrich) and [9,10-^3^H] oleic acid (Perkin Elmer, Waltham, MA, USA), 0.5 µCi per sample. The ^3^H_2_O released from tritium-labeled oleic acid was captured with a Dowex-OH^−^ resin (1 × 8–200; Sigma-Aldrich), eluted with water into scintillation vials, and quantified using liquid scintillation counting. The results were normalized to protein concentration.

Citrate synthase (CS) activity was measured in differentiated C2C12 cells as described previously [26] and normalized to protein concentration. Assessment of mitochondrial respiration in differentiated C2C12 myotubes was performed using a Seahorse XF96 extracellular flux analyzer (Agilent Technologies, Santa Clara, CA, USA). Cellular oxygen consumption rate (OCR) was measured under basal conditions and in the presence of 2 µM mitochondrial uncoupler carbonyl cyanide 4-(trifluoromethoxy) phenylhydrazone (FCCP) (Abcam, Cambridge, UK) to assess maximal respiration. The results were normalized to protein concentration.

### Statistical analysis

Data were analyzed using Prism 6.0 software (GraphPad, San Diego, CA, USA) and presented as mean ± SEM. One-way ANOVA with Bonferroni adjustment for multiple comparisons was used to calculate differences between groups. Unpaired two-tailed *t*-test was performed for comparison of two groups. *P* value of <0.05 was assigned to be statistically significant. During the experiment, one control *db/+* mouse and two control *db/db* mice encountered premature death. In addition, two *db/db* mice suffered of malocclusion and were excluded from the data analysis due to severe weight loss.

## Results

### TNKS inhibition reduces body weight gain and fat mass and alleviates dyslipidemia in *db/db* mice

**Fig. 1:**
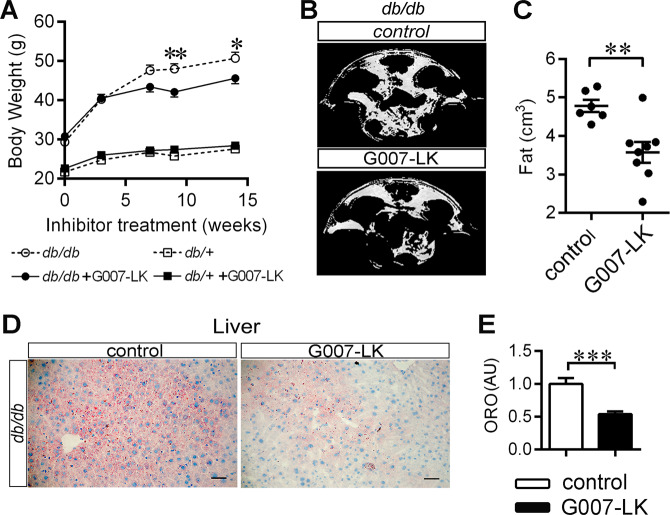
TNKS inhibition reduces body weight gain, fat mass, and hepatic steatosis. **a** Male 6 weeks old diabetic *db/db* and nondiabetic *db/+* mice were fed either with regular chow or chow spiked with G007-LK for 15 weeks. Body weight was monitored at indicated time points. **b**, **c** MRI analysis at 9 weeks of treatment for the abdominal fat volume of G007-LK-treated and nontreated *db/db* mice. **b** Representative MRI images showing fat distribution (white color). The spine is at the top of the image. **c** MRI-based calculation of abdominal fat volume. **d**, **e** Oil Red O (ORO) staining at 15 weeks of treatment analyzing hepatic steatosis of G007-LK treated and control *db/db* mice. **d** Representative images of liver sections of *db/db* mice (Scale bar, 50 µm). **e** Graph represents quantification of the ORO staining intensity (AU, arbitrary unit). Error bars represent ±SEM. One-way ANOVA with Bonferroni correction for multiple comparisons, two-tailed *t*-test. *p < 0.05; **p < 0.01. In (**a**), *db/db n* = 6; *db/db* + G007-LK *n* = 10; *db/+ n* = 9; *db/+* + G007-LK *n* = 10. In (**b**, **c**), control *n* = 6; G007-LK *n* = 8. In (**e**), *n* = 4 in each group.

Diabetic *db/db* and nondiabetic *db/+* male mice at the age of six weeks received either normal chow or chow supplemented with a selective TNKS inhibitor G007-LK. After 15 weeks of G007-LK treatment, neither genotype exhibited mortality or inhibition of intestinal epithelial cell proliferation within the crypts in comparison with nontreated ones (Fig. S1), indicating that G007-LK is not harmful at the given dosage. G007-LK treatment attenuated weight gain (Fig. 1a), decreased abdominal adiposity (by 25%, Fig. 1b, c), decreased steatosis (Fig. 1d, e), and led to a trend of decreased adipocyte cell size in WAT (Fig. S2) without affecting food intake (Fig. S3A), wheel-running activity (Fig. S3B, C), or core body temperature (Fig. S3D) in *db/db* mice. G007-LK treatment alleviated dyslipidemia of the *db/db* mice by lowering serum low-density lipoprotein cholesterol, high-density lipoprotein cholesterol, and total cholesterol (Table 1), whereas with triglycerides, glycerol, and nonesterified fatty acids the difference between treated and nontreated groups (Table 1) did not reach statistical significance. In contrast to *db/db* mice, G007-LK had no effect on body weight or serum lipids in nondiabetic *db/+* mice (Fig. 1 and Table 1). Fasting blood glucose, serum insulin levels, or insulin tolerance test did not show obvious difference between G007-LK-treated and nontreated *db/db* mice (Fig. S3E–H).

**Table 1:** Serum lipid profile of *db/db* and *db/+* mice after 15 weeks of G007-LK treatment.

	*db/db*		*db/+*	
	Control *n* = 5 (4)	G007-LK *n* = 8 (6)	Control *n* = 9 (6)	G007-LK *n* = 9 (7)
TC (mmol/l)	2.68 ± 0.25^###^	1.95 ± 0.14**	1.74 ± 0.08	1.91 ± 0.10
LDL (mmol/l)	0.18 ± 0.04^#^	0.08 ± 0.01**	0.11 ± 0.01	0.10 ± 0.01
HDL (mmol/l)	0.92 ± 0.03^##^	0.71 ± 0.06*	0.62 ± 0.03	0.71 ± 0.04
TG (mmol/l)	1.78 ± 0.29^###^	1.66 ± 0.14	0.89 ± 0.09	0.76 ± 0.05
Glycerol (mmol/l)	0.31 ± 0.02^###^	0.26 ± 0.03	0.17 ± 0.01	0.18 ± 0.02
NEFA (mmol/l)	1.18 ± 0.19^#^	0.85 ± 0.12	0.58 ± 0.07	0.66 ± 0.09

Mean ± SEM, *n* numbers for measuring glycerol are indicated in parenthesis.

*TC* total cholesterol, *LDL* low-density lipoprotein cholesterol, *HDL* high-density lipoprotein cholesterol, *TG* triglycerides, *NEFA* nonesterified fatty acids.

*p < 0.05; **p < 0.01 vs. nontreated mice in each group, ^*###*^*p* < 0.001; ^##^*p* < 0.01; ^#^*p* < 0.05 vs. *db/+*.

### G007-LK induces AXIN1 accumulation in WAT and muscle in *db/db* mice

To confirm the effectiveness of G007-LK, we defined the expression levels of TNKS1/2 and AXIN1 in WAT and muscle. AXIN1 is targeted for proteolysis by TNKSs and thereby TNKS inhibition stabilizes AXIN1 [27]. Both TNKS1 and TNKS2 are expressed in WAT in *db/db* and *db/+* mice (Fig. S4A), migrating at 160 kDa and 130 kDa, respectively, as previously demonstrated by transient overexpression in HEK293 cells [20]. G007-LK reduced the protein level of TNKS1 and TNKS2 in WAT in both genotypes (Fig. S4A). Furthermore, AXIN1 accumulated in WAT in G007-LK-treated *db/db* mice (Fig. S4B). This indicates that G007-LK diminished TNKS activity in WAT. Only TNKS1 protein was detected in muscle (Fig. S4C). As in WAT, G007-LK diminished the protein level of TNKS1 in muscle in both genotypes (Fig. S4C) and induced AXIN1 accumulation in *db/db* mice (Fig. S4D).

### TNKS inhibition suppresses proteins involved in lipolysis, induces beiging, and upregulates adiponectin in WAT in *db/db* mice

**Fig. 2:**
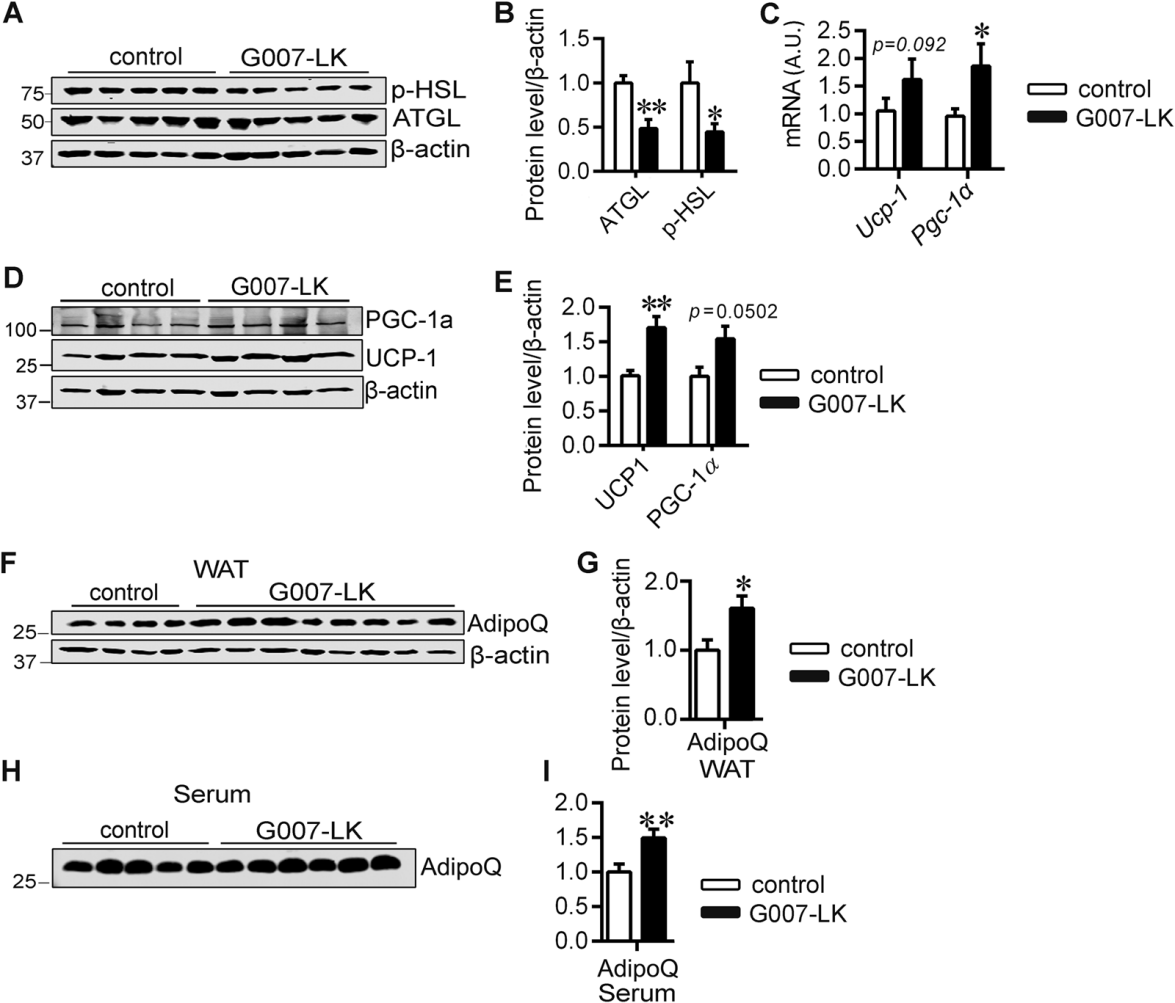
TNKS inhibition restrains lipolysis-associated proteins, induces browning of WAT and increases adiponectin level in WAT, and serum in *db/db* mice. Representative immunoblot (**a**) and quantification (**b**) of ATGL and phospho-HSL in WAT of G007-LK-treated and control mice. **c** qRT-PCR analyses of *UCP-1* and *PGC-1α* transcripts in WAT of *db/db* mice (A.U., arbitrary unit). Representative immunoblot (**d**) and quantification (**e**) of UCP-1 and PGC-1α in WAT of G007-LK-treated and control *db/db* mice. **f**–**i** Representative immunoblots (**f**, **h**) and corresponding quantifications (**g**, **i**) of adiponectin (AdipQ) in WAT (**f**, **g**) and serum (**h**, **i**) of G007-LK-treated and control mice after 15 weeks of treatment. The graphs represent quantifications of the indicated proteins normalized to actin (**b**, **e**, **g**, and **l**) or quantification of the individual bands on the blots (**i**). Error bars represent ±SEM. One-way ANOVA with Bonferroni adjustment for multiple comparisons, two-tailed *t*-test. *p < 0.05; **p < 0.01. In WAT (**a**–**g**), control *n* = 4–7; G007-LK *n* = 5–8, in serum (**h**, **i**), control *n* = 5; G007-LK *n* = 6.

To investigate the mechanism of the observed weight loss and improvement of dyslipidemia, we analyzed the effect of G007-LK-treatment on molecules associated with lipid metabolism in WAT. G007-LK reduced the protein levels of lipolysis-related adipose triglyceride lipase (ATGL) and Ser660-phosphorylated hormone sensitive lipase (p-HSL, active form) in *db/db* mice (Fig. 2a, b). TNKS inhibition did not affect ATGL and p-HSL in WAT of *db/+* mice (Fig. S5A, B). These data together suggest that TNKS inhibition suppresses molecules associated with lipolysis in WAT of *db/db* mice.

G007-LK did not alter lipid synthesis, indicated by the unaltered protein levels of fatty acid synthase or Ser79-phosphorylated-acetyl-CoA carboxylase (inactive form) in WAT in either *db/db* or *db/+* mice (Fig. S6A). Nor did it affect the mRNA levels of transcription factors sterol regulatory element binding protein 1 (*Srebp1*) and *Pparγ*, the master regulators of lipogenesis and adipogenesis (Fig. S6B), or the expression level of fatty acid translocase/cluster of differentiation 36 (*Fat/Cd36*) (Fig. S6B), a target gene of PPARγ that is involved in fatty acid uptake [28]. Interestingly, inhibition of TNKSs stimulated the expression of uncoupling protein 1 (UCP-1), a marker of brown and beige adipocytes [29], at the protein level (Fig. 2d, e) without an increase of its mRNA level (Fig. 2c). In addition, TNKS inhibition increased the level of *Pgc-1α* transcript (Fig. 2c) and tended to boost PGC-1α protein level (Fig. 2d, e), which in complex with PPARγ binds to the *Ucp-1* promoter to activate UCP-1 expression [30]. Furthermore, inhibition of TNKS activity upregulated adiponectin, an endogenous insulin sensitizer and a stimulator of FAO [31], in both WAT (Fig. 2f, g) and serum (Fig. 2h, i) of *db/db* mice. The increase of adiponectin was not observed in TNKS inhibitor-treated *db*/+ mice (Fig. S5C–F).

### TNKS inhibition has no effect on genes associated with lipid utilization or gluconeogenesis in liver of *db/db* mice

Both TNKSs were detected in the liver of *db/db* and *db/+* mice and G007-LK-treatment reduced TNKS expression in *db/db* mice (Fig. S7A). However, the liver content of AXIN1 was not significantly increased by G007-LK treatment in *db/db* mice (Fig. S7B), indicating that G007-LK does not inhibit TNKS activity, as measured by AXIN1 accumulation, in liver. We further defined whether decreased lipolytic flux from WAT to liver has generated metabolic signals that modulate lipid metabolism and gluconeogenesis in liver. We observed that TNKS inhibition had no effect on genes involved in the transcriptional regulation of lipid oxidation (no difference in *Srebp1*, *Pparα* expression), FAO and uptake (no difference in acyl-CoA oxidase 1 (*Aco1*)*, Fat/Cd36*, and carnitine palmitoyltransferase-1 beta (*Cpt1β*) expression) or gluconeogenesis (no difference in *G6pc* and *Pck1* expression) in liver of *db/db* mice (Fig. S7C). Notably, G007-LK treatment did not alter the transcript or the protein levels of PGC-1α in liver in *db/db* mice (Fig. S7C, D).

### TNKS inhibition increases muscle mitochondrial mass and oxidative metabolism

**Fig. 3:**
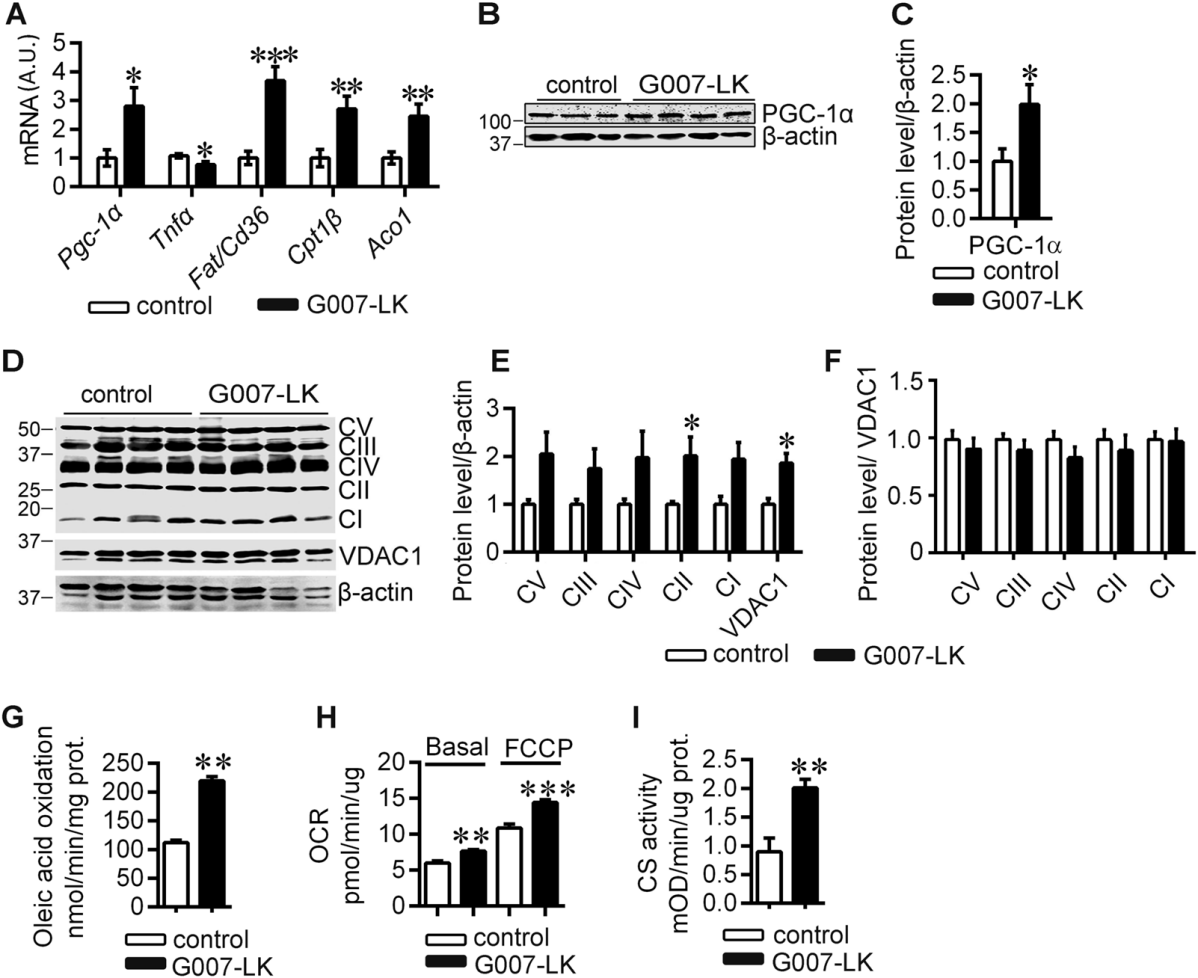
TNKS inhibition increases mitochondrial mass in muscle in *db/db* mice and mitochondrial mass and oxidative metabolism in cultured myotubes. **a** qRT-PCR analyses of genes associated with fatty acid oxidation in muscle of G007-LK-treated and control *db/db* mice (A.U., arbitrary unit). Representative immunoblot (**b**) and quantification (**c**) of PGC-1α in muscle of G007-LK-treated and control *db/db* mice. Immunoblot analysis (**d**) and corresponding quantification (**e**, **f**) of the subunits of mitochondrial OXPHOS complexes and VDAC1 normalized to β-actin (**e**) and the OXPHOS complexes normalized to VDAC1 (**f**) in G007-LK treated and control *db/db* muscle. CI: NDUFB8, (NADH dehydrogenase 1β subcomplex subunit 8), CII: SDHB, (succinate dehydrogenase b iron–sulfur subunit), CIII: UQCRC2 (cytochrome b-c1 complex subunit 2), CIV: MTCO1 (cytochrome c oxidase subunit 1) and CV: ATP5A (ATP synthase subunit α). Oleic acid oxidation (**g**), O_2_ consumption rates (OCR) (**h**), and citrate synthase activity (**i**) in C2C12 myotubes after 24 h pretreatment with either vehicle or 10 nM G007-LK, the lowest effective concentration. Error bars represent ±SEM. Two-tailed *t*-test and in (**g**–**i**), two-way ANOVA for multiple comparisons. *p < 0.05; **p < 0.01; ***p < 0.001. In muscle (**a**–**f**) control *n* = 3–5; G007-LK *n* = 4–5. In oleic acid oxidation (**g**) control *n* = 4; G007-LK *n* = 6, in Seahorse respirometry (**h**) control *n* = 10; G007-LK *n* = 20, and in citrate synthase assay (**i**) control *n* = 10; G007-LK *n* = 20. Assays were repeated several times in C2C12 myotubes and representative results are shown from one batch of cells after G007-LK treatment.

The improved lipid profile and reduced adiposity of the TNKS inhibitor-treated *db/db* mice led us to investigate molecules associated with lipid oxidative metabolism in muscle. TNKS inhibition upregulated both *Pgc-1α* transcript (Fig. 3a) and protein (Fig. 3b, c) in muscle in *db/db* mice. Notably, *Tnfα*, a mediator of metabolic disorders [32] that inhibits PGC-1α expression [33], was downregulated by G007-LK treatment in muscle of *db/db* mice (Fig. 3a). In line with the upregulation of PGC-1α, TNKS inhibition upregulated genes involved in fatty acid uptake, *Fat/Cd36*, and β-oxidation, *Cpt1β* and *Aco1* (Fig. 3a), suggesting enhanced FAO. The genes involved in fatty acid uptake and oxidation were not affected by TNKS inhibitor treatment in muscle of *db/+* mice (Fig. S8).

We further investigated the possible effects of PGC-1α upregulation upon TNKS inhibition on mitochondrial biogenesis and oxidative phosphorylation (OXPHOS). TNKS inhibition significantly increased the protein amount of SDHB (Fig. 3d, e), a mitochondrial complex II (CII) subunit protein that functions in both the TCA-cycle and the electron transport chain [34], and the other OXPHOS complex subunits tended to increase (Fig. 3d, e). In addition, TNKS inhibition increased mitochondrial outer membrane protein VDAC1 expression (Fig. 3d, e), indicative of upregulation of mitochondrial mass. In order to define whether there was a difference in the levels of OXPHOS complex subunits per mitochondrion, the amount of OXPHOS complex subunit proteins was normalized to VDAC1. These data did not reveal differences between the inhibitor-treated and nontreated mice (Fig. 3f), suggesting that TNKS inhibition elevated mitochondrial mass in the muscle of *db/db* mice.

To confirm the findings in muscle tissue we analyzed the effect of TNKS inhibitor treatment on FAO and mitochondrial function and amount in C2C12 myotubes. We observed that G007-LK stimulated oleic acid oxidation in myotubes (Fig. 3g). G007-LK also boosted basal and maximum OCR in C2C12 myotubes (Fig. 3h), suggesting improved mitochondrial function. Given that G007-LK enhanced CS activity in myotubes (Fig. 3i), the indicator of mitochondrial content, the observed increase in mitochondrial respiration is most likely caused, at least partially, by higher mitochondrial mass. These data collectively suggest that G007-LK treatment enhances mitochondrial mass and oxidative metabolism in muscle and myotubes.

### TNKSs interact with and PARylate PGC-1α in WAT and muscle

**Fig. 4:**
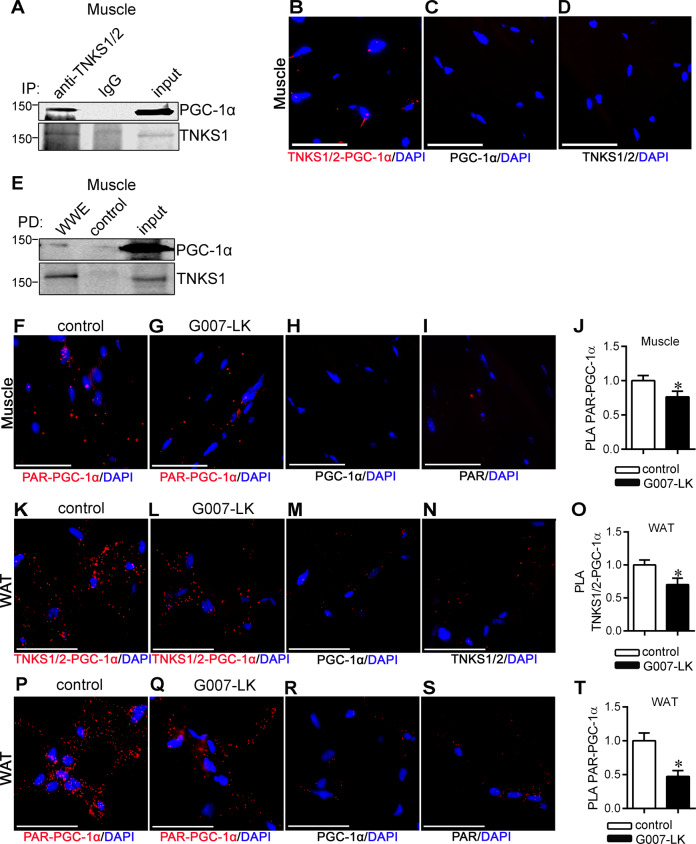
TNKS inhibition reduces PGC-1α PARylation in muscle and WAT of *db/db* mice. **a** Immunoprecipitation of muscle lysates from normal mice with TNKS1/2 antibody and rabbit IgG as a control. Fractions of the immunoprecipitates were separated in individual lanes and immunoblotted with anti-TNKS1/2 and anti-PGC-1α IgGs. **b–d** Duolink in situ PLA showing association of TNKS1 with PGC-1α in muscle of control *db/db* mice. Representative image of TNKS1/2-PGC-1α association (**b**) and PLA performed with either anti-PGC-1α IgG (**c**) or anti-TNKS1/2 IgG (**d**) alone. Scale bar, 50 μm. **e** Pull-down assay of muscle lysate from normal mice with PAR-affinity resin (WWE) followed by immunoblotting with anti-PGC-1α or anti-TNKS1/2. Control: negative control resin harboring a mutation in WWE that abolishes PAR binding. **f**–**j** Duolink in situ PLA showing PARylation of PGC-1α in muscle of G007-LK-treated and control *db/db* mice. Representative image of PARylated PGC-1α in muscle in control (**f**) and G007-LK-treated (**g**) *db/db* mice. PLA performed with either anti-PGC-1α IgG (**h**) or anti-PAR IgG (**i**) alone. Quantification of PGC-1α PARylation level (**j**). Scale bar, 50 µm. **k**–**o** Duolink in situ PLA showing association of TNKS1 with PGC-1α in WAT of G007-LK-treated and control *db/db* mice. Representative image of TNKS1/2-PGC-1α association in control (**k**) and G007-LK-treated *db/db* mice (**i**). PLA performed with either anti-PGC-1α IgG (**m**) or anti-TNKS1/2 IgG (**n**) alone. Quantification of PGC-1α and TNKS1/2 association (**o**). Scale bar, 50 μm. **p**–**t** Duolink in situ PLA showing PARylation of PGC-1α in WAT of G007-LK-treated and control *db/db* mice. Representative image of PARylated PGC-1α in WAT in control (**p**) and G007-LK-treated (**q**) *db/db* mice. PLA performed with either anti-PGC-1α IgG (**r**) or anti-PAR IgG (**s**) alone. Quantification of PGC-1α PARylation level (**t**). Scale bar, 50 µm. Error bars represent ±SEM. Two-tailed *t*-test. *p < 0.05. In muscle (**a**–**j**) *n* = 3 in each group, in WAT (**k**–**t**) *n* = 4 for each group.

The upregulation of PGC-1α in muscle and WAT in *db/db* mice upon TNKS inhibition prompted us to explore the possible mechanisms. We found that TNKS1/2 co-immunoprecipitate with PGC-1α from muscle lysate of normal mice (Fig. 4a). PLA confirmed that PGC-1α and TNKS1/2 form a complex in muscle tissue of *db/db* mice (Fig. 4b–d). Moreover, pull-down with PAR-affinity resin from the muscle lysate of normal mice followed by immunoblotting for PGC-1α revealed that PGC-1α is PARylated (Fig. 4e). PLA analysis of PARylation of PGC-1α indicated that TNKS inhibition reduces PARylation of PGC-1α in muscle (Fig. 4f–j). PLA analysis also revealed that PGC-1α associates with TNKS1/2 (Fig. 4k–n) and is PARylated (Fig. 4p–s) in WAT. Moreover, both the association of TNKSs with PGC-1α (Fig. 4o) and the PARylation of PGC-1α (Fig. 4t) in WAT were reduced upon TNKS inhibition. Collectively, the data indicate that TNKSs form a complex with and PARylate PGC-1α in WAT and muscle.

## Discussion

**Fig. 5:**
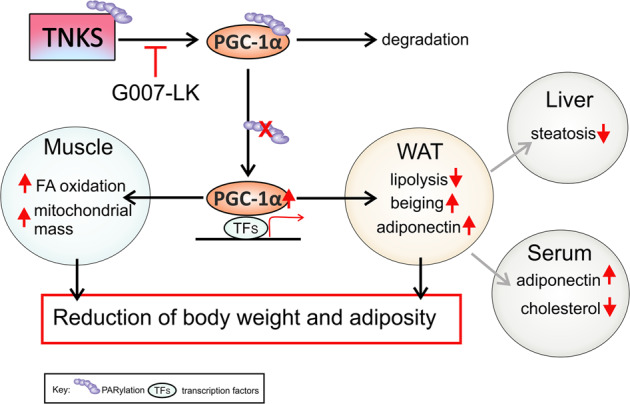
Schematic cartoon visualizing the effects of the inhibition of TNKS-mediated PARylation on PGC-1α and the metabolic consequences. PGC-1α is PARylated by TNKSs in adipose tissue and muscle and targeted for degradation. G007-LK inhibits the PARylation activity of TNKSs and, thereby, reduces the PARylation of PGC-1α, stabilizing its expression. This turns on specific PGC-1α-mediated transcriptional activity, leading to beneficial tissue-specific downstream effects: increase of mitochondrial mass and fatty acid (FA) oxidation in muscle, reduction of lipolysis-associated proteins ATGL and active HSL, increase of beiging-associated UCP-1 protein in adipose tissue, and upregulation of adiponectin in adipose tissue and serum. G007-LK treatment also ameliorates liver steatosis and downregulates serum cholesterol. These mechanisms contribute to the subsequent reduction of body weight gain and adiposity.

In this study we demonstrate that inhibition of TNKS activity prevents body weight gain, restrains fat accumulation, and alleviates dyslipidemia. We further show that TNKS inhibition increases adiponectin, suppresses proteins associated with lipolysis and induces browning of WAT. TNKS inhibition also stimulates lipid oxidation in muscle and decreases liver steatosis. A key finding in the study is that TNKS inhibition in *db/db* mice leads to reduced TNKS-derived PARylation and the consequent increase of the expression of PGC-1α, the central transcriptional regulator of energy metabolism, in WAT and muscle (Fig. 5).

The transcriptional activity and metabolic functions of PGC-1α are modulated by posttranslational modifications [6], which include phosphorylation, ubiquitination, methylation, acetylation, and GlcNAcylation [35–39]. Our study unveils PARylation as a novel posttranslational mechanism whereby PGC-1α is regulated by TNKSs in a tissue-specific manner (Fig. 5). We demonstrate that TNKS1/2 forms a physiological complex with PGC-1α in muscle and WAT of *db/db* mice, and that TNKS inhibition attenuates the PARylation of PGC-1α. This could prevent the proteolytic degradation of PGC-1α and consequent induction of a metabolic gene transcription program beneficial for the metabolic health in muscle and WAT (Fig. 5). Notably, PGC-1α exerts opposing effects on metabolic pathways in a tissue-specific manner. Consequently, whole body overexpression of PGC-1α increases hepatic insulin resistance and gluconeogenesis but improves muscle insulin sensitivity [40]. This creates a major challenge in targeting PGC-1α to treat metabolic disorders [5] since tissue-selective manipulation of PGC-1α activity may be required for maximal efficacy. In line with the unaltered TNKS activity in liver upon G007-LK treatment, the expression of PCG-1α and genes responsible for FAO and gluconeogenesis remained unchanged in liver, whereas PGC-1α was upregulated in WAT and muscle improving metabolic health. Thus, our study suggests that pharmacological inhibition of TNKSs may be a viable strategy to stimulate PGC-1α selectively in WAT and muscle without affecting its hepatic activity. In addition, our finding advances mechanistic insights by which TNKSs regulate cellular metabolism and provides a novel mechanism, TNKS-mediated PARylation, for regulating the metabolic activity of PGC-1α.

G007-LK has been explored in the treatment of cancer as it attenuates WNT/β-catenin signaling [14]. Even though the study by Lau et al. raised a concern of potential intestinal toxicity of the compound when administered at high dosage, apparently due to reduced intestinal crypt cell proliferation that is regulated by Wnt signaling and TNKSs [14], and another study reported bone loss in mice [15], other studies, including this study, have found the inhibitor well tolerated without any effects on intestinal structure or function [41, 42]. In metabolic studies lasting 15 weeks (this study) and 6 months [18], no intestinal pathology or any other signs of toxicity were observed. In contrast, we documented marked beneficial metabolic effects. Thus, our work opens a new avenue proposing that drugs targeting TNKSs tested at present in cancer field may be retooled to treat metabolic disorders without adverse effects.

We observed improved lipid oxidative metabolism coupled with reduction of body weight gain and abdominal fat mass in *db/db* mice after pharmacological TNKS inactivation. Similar to these observations, both global deficiency and adipocyte-selective inactivation of TNKS1 lead to reduced fat mass [17, 18]. A recent publication by Li et al. showed improved glycemic control after administering 30 mg G007-LK/kg/day for 30 days, five times a week [43]. In contrast, in our study the inhibitor treatment (14 mg G007-LK/kg/day) did not reduce blood glucose, suggesting that a high dose of G007-LK may be required for achieving better glycemic control in *db/db* mice. On the other hand, it has been shown in vitro in L6 myocytes, that TNKS inhibition results in reduced insulin stimulated glucose uptake [44]. If TNKS inhibition would increase insulin resistance in a similar manner in vivo, this could counteract the positive effect of weight loss on insulin sensitivity, though this contradicts with the study by Li et al. [43]. The effects of TNKS inhibition on glucose uptake appear to be cell-type specific, as TNKS inhibition has been shown to enhance glucose uptake into adipocytes [45]. Nevertheless, these data together suggest that TNKS inactivation provides a potential approach to improve lipid and glucose metabolism in obesity and diabetes.

Our data demonstrate that TNKS inhibition in diabetic mice may repress lipolysis in WAT as protein levels of active HSL and ATGL were reduced. Excessive lipolysis is evident in adults with T2DM [46] and in obese youth with impaired glucose tolerance [47]. Unrestrained lipolysis in WAT results in ectopic lipid accumulation and contributes to the development of insulin resistance in obese individuals [46, 48–50]. Thus, suppression of lipolysis in WAT could be an attractive approach to prevent or treat insulin resistance in obesity and T2DM. Reduction of lipolysis is in line with the observation of ameliorated hepatic steatosis in G007-LK-treated *db/db* mice. In addition, adiponectin, that has been shown to reduce hepatic steatosis [51], was upregulated in WAT and serum in *db/db* mice treated with G007-LK, similarly to global TNKS1 deficient mice [17].

We observed that TNKS inhibition induces upregulation of UCP-1, specific for brown adipose tissue, in *db/db* mice, indicating beiging of WAT. Supporting our data in mice, a previous in vitro study using primary mouse adipocytes revealed that TNKS inhibition by XAV939 increases UCP-1 expression [52]. UCP-1 is crucial to brown and beige adipocyte function and beiging of WAT represents an important approach to alleviate dyslipidemia [30, 53]. This is based on the observation that mice lacking UCP-1 gain weight when kept at thermoneutral conditions [54], while transgenic expression of UCP-1 in adipose tissue reduces body weight and fat mass in mice [55]. In line with this, TNKS inhibition-induced beiging of WAT might contribute to the reduction of weight gain and abdominal fat in *db/db* mice in the current study. From a mechanistic point of view, UCP-1 expression is regulated by the PGC-1α-PPARγ complex [30], which binds to the UCP-1 promoter to activate UCP-1 expression. Therefore, the accumulation of UCP-1 may reflect the increase of PGC-1α expression upon TNKS inhibition.

We found elevated expression of genes involved in mitochondrial FAO in muscle in *db/db* mice treated with G007-LK. This is consistent with the previous studies showing that pan-PARP inhibitor treatment and genetic ablation of PARP-1 promotes FAO and conserves mitochondrial function in muscle [56, 57]. Enhanced lipid oxidation was also observed in muscle of mice with global but not adipocyte-specific deficiency of TNKS1 [17, 18]. We further observed that TNKS inhibition with G007-LK stimulates FAO and increases mitochondrial respiration and amount in C2C12 myotubes, suggesting that TNKS inhibition enhances mitochondrial fatty acid oxidative capacity in muscle. Contrary to this, genetic deletion on TNKS did not affect respiration rates in mitochondria isolated from muscle [17]. The difference could be due to different experimental approaches, isolated mitochondria versus mitochondria in intact cells.

As we observed no obvious increase in food intake or reduction in physical activity, which could contribute to the loss of body weight, our data raised an interesting question whether enhanced lipid oxidation in muscle, together with enhanced beiging of WAT, could contribute to the reduction of abdominal fat mass and body weight upon TNKS inhibition. Two previous studies, one on Pima Indians and the other on lean women, revealed that low capacity to oxidize fatty acids associates with increased risk of weight gain [58, 59]. It is thus plausible that TNKS inhibition could contribute to the reduction of body weight by enhancing FAO in muscle. Consequently, due to the evident interorgan crosstalk, adipose tissue-derived adiponectin may further enhance FAO in muscle [31] boosting up the positive metabolic outcome of TNKS inhibition. The increase of PGC-1α and UCP-1, both regulators of thermogenesis [60, 61], in muscle and adipose tissue of TNKS inhibitor-treated *db/db* mice suggests that TNKS inhibition could lead to an increase in thermogenesis. To conclusively indicate that TNKS inhibition leads to increased mitochondrial uncoupling and energy expenditure, contributing to the loss of body weight, remains to be clarified in further studies. Combining TNKS inhibition with increased physical activity could further boost the beneficial metabolic effects via PGC-1α, as a previous study revealed that overexpression of PGC-1α in muscle leads to insulin resistance of sedentary mice receiving high fat diet, but exercise leads to improved insulin sensitivity in these mice [62].

Taken together, we demonstrate that pharmacological inhibition of TNKS activity increases the expression of the transcriptional coactivator PGC-1α in WAT and muscle. This induces browning of WAT, increases adiponectin levels in WAT and serum and improves mitochondrial mass and fatty acid metabolism in muscle of *db/db* mice (Fig. 5). Most importantly, this occurs without increasing the expression of PGC-1α in liver, avoiding the undesirable increase in gluconeogenesis. These data highlight the potential of inhibiting TNKSs as a pharmacotherapy for obesity and T2DM.

## Supplementary information

Wang et al. Supplemental material

## References

[1] Rosen ED, Spiegelman BM (2014). What we talk about when we talk about fat. Cell.

[2] Kelley DE, Simoneau JA (1994). Impaired free fatty acid utilization by skeletal muscle in non-insulin-dependent diabetes mellitus. J Clin Investig.

[3] Mogensen M, Sahlin K, Fernström M, Glintborg D, Vind BF, Beck-Nielsen H (2007). Mitochondrial respiration is decreased in skeletal muscle of patients with type 2 diabetes. Diabetes.

[4] Hesselink MKC, Schrauwen-Hinderling V, Schrauwen P (2016). Skeletal muscle mitochondria as a target to prevent or treat type 2 diabetes mellitus. Nat Rev Endocrinol.

[5] Rowe G, Arany Z (2014). Genetic models of PGC-1 and glucose metabolism and homeostasis. Rev Endocr Metab Disord.

[6] Fernandez-Marcos PJ, Auwerx J (2011). Regulation of PGC-1α, a nodal regulator of mitochondrial biogenesis. Am J Clin Nutr.

[7] Puigserver P, Rhee J, Donovan J, Walkey CJ (2003). Insulin-regulated hepatic gluconeogenesis through FOXO1-PGC-1Alpha interaction. Nature.

[8] Finck BN, Kelly DP (2006). PGC-1 coactivators: inducible regulators of energy metabolism in health and disease. J Clin Investig.

[9] Rhee J, Donovan J, Yoon JC, Newgard CB, Puigserver P, Stafford J (2001). Control of hepatic gluconeogenesis through the transcriptional coactivator PGC-1. Nature.

[10] Mensink M, Hesselink MKC, Russell AP, Schaart G, Sels J, Schrauwen P (2007). Improved skeletal muscle oxidative enzyme activity and restoration of PGC-1α and PPARβ/δ gene expression upon rosiglitazone treatment in obese patients with type 2 diabetes mellitus. Int J Obes.

[11] Semple RK, Crowley VC, Sewter CP, Laudes M, Christodoulides C, Considine RV (2004). Expression of the thermogenic nuclear hormone receptor coactivator PGC-1α is reduced in the adipose tissue of morbidly obese subjects. Int J Obes.

[12] Yoon JC, Puigserver P, Chen G, Donovan J, Wu Z, Rhee J (2001). Control of hepatic gluconeogenesis through the transcriptional coactivator PGC-1. Nature.

[13] Smith S, Giriat I, Schmitt A, de Lange T (1998). Tankyrase, a poly(ADP-ribose) polymerase at human telomeres. Science.

[14] Lau T, Chan E, Callow M, Waaler J, Boggs J, Blake RA (2013). A novel tankyrase small-molecule inhibitor suppresses APC mutation-driven colorectal tumor growth. Cancer Res.

[15] Fujita S, Mukai T, Mito T, Kodama S, Nagasu A, Kittaka M (2018). Pharmacological inhibition of tankyrase induces bone loss in mice by increasing osteoclastogenesis. Bone.

[16] Yeh TY, Sbodio JI, Tsun ZY, Luo B, Chi NW (2007). Insulin-stimulated exocytosis of GLUT4 is enhanced by IRAP and its partner tankyrase. Biochem J.

[17] Yeh TY, Beiswenger KK, Li P, Bolin KE, Lee RM, Tsao TS (2009). Hypermetabolism, hyperphagia, and reduced adiposity in tankyrase-deficient mice. Diabetes.

[18] Zhong L, Ding Y, Bandyopadhyay G, Waaler J, Börgeson E, Smith S (2016). The PARsylation activity of tankyrase in adipose tissue modulates systemic glucose metabolism in mice. Diabetologia.

[19] Eisemann T, Langelier M, Pascal JM (2019). Structural and functional analysis of parameters governing tankyrase-1 interaction with telomeric repeat-binding factor 1 and GDP-mannose dehydratase. J Biol Chem.

[20] Kuusela S, Wang H, Wasik AA, Suleiman H, Lehtonen S (2016). Tankyrase inhibition aggravates kidney injury in the absence of CD2AP. Cell Death Dis.

[21] Pezzolesi MG, Nam M, Nagase T, Klupa T, Dunn JS, Mlynarski WM (2004). Examination of candidate chromosomal regions for type 2 diabetes reveals a susceptibility locus on human chromosome 8p23.1. Diabetes.

[22] Scherag A, Dina C, Hinney A, Vatin V, Scherag S, Vogel CIG (2010). Two new loci for body-weight regulation identified in a joint analysis of genome-wide association studies for early-onset extreme obesity in French and German study groups. PLoS Genet.

[23] Wang H, Semenova S, Kuusela S, Panula P, Lehtonen S (2015). Tankyrases regulate glucoregulatory mechanisms and somatic growth via the central melanocortin system in zebrafish larvae. FASEB J.

[24] Lehtonen S, Lehtonen E, Kudlicka K, Holthöfer H, Farquhar MG (2004). Nephrin forms a complex with adherens junction proteins and CASK in podocytes and in Madin-Darby canine kidney cells expressing nephrin. Am J Pathol.

[25] Chegary M, Brinke HT, Ruiter JPN, Wijburg FA, Stoll MSK, Minkler PE (2009). Mitochondrial long chain fatty acid β-oxidation in man and mouse. BBA-Mol Cell Biol Lipids.

[26] Trounce IA, Kim YL, Jun AS, Wallace DC (1996). Assessment of mitochondrial oxidative phosphorylation in patient muscle biopsies, lymphoblasts, and transmitochondrial cell lines. Methods Enzymol.

[27] Huang SM, Mishina YM, Liu S, Cheung A, Stegmeier F, Michaud GA (2009). Tankyrase inhibition stabilizes axin and antagonizes Wnt signalling. Nature.

[28] Holloway GP, Luiken JJ, Glatz JF, Spriet LL, Bonen A (2008). Contribution of FAT/CD36 to the regulation of skeletal muscle fatty acid oxidation: an overview. Acta Physiol.

[29] Wu J, Bostrom P, Sparks LM, Ye L, Choi JH, Giang AH (2012). Beige adipocytes are a distinct type of thermogenic fat cell in mouse and human. Cell.

[30] Bartelt A, Heeren J (2014). Adipose tissue browning and metabolic health. Nat Rev Endocrinol.

[31] Fruebis J, Tsao TS, Javorschi S, Ebbets-Reed D, Erickson MR, Yen FT (2001). Proteolytic cleavage product of 30-kDa adipocyte complement-related protein increases fatty acid oxidation in muscle and causes weight loss in mice. Proc Natl Acad Sci USA.

[32] Hotamisligil GS, Spiegelman BM (1994). Tumor necrosis factor alpha: a key component of the obesity-diabetes link. Diabetes.

[33] Tang K, Wagner PD, Breen EC (2010). TNF-alpha-mediated reduction in PGC-1alpha may impair skeletal muscle function after cigarette smoke exposure. J Cell Physiol.

[34] Bezawork-Geleta A, Rohlena J, Dong L, Pacak K, Neuzil J (2017). Mitochondrial complex II: at the crossroads. Trends Biochem Sci.

[35] Lerin C, Rodgers JT, Kalume DE, Kim S, Pandey A, Puigserver P (2006). GCN5 acetyltransferase complex controls glucose metabolism through transcriptional repression of PGC-1α. Cell Metab.

[36] Gerhart-Hines Z, Rodgers JT, Bare O, Lerin C, Kim S-, Mostoslavsky R (2007). Metabolic control of muscle mitochondrial function and fatty acid oxidation through SIRT1/PGC-1[alpha]. EMBO J.

[37] Anderson RM, Barger JL, Edwards MG, Braun KH, O’Connor CE, Prolla TA (2008). Dynamic regulation of PGC‐1α localization and turnover implicates mitochondrial adaptation in calorie restriction and the stress response. Aging Cell.

[38] Teyssier C, Ma H, Emter R, Kralli A, Stallcup MR (2005). Activation of nuclear receptor coactivator PGC-1alpha by arginine methylation. Genes Dev.

[39] Jäger S, Handschin C, St.-Pierre J, Spiegelman BM (2007). AMP-activated protein kinase (AMPK) action in skeletal muscle via direct phosphorylation of PGC-1α. Proc Natl Acad Sci USA.

[40] Liang H, Balas B, Tantiwong P, Dube J, Goodpaster BH, O’Doherty RM (2009). Whole body overexpression of PGC-1α has opposite effects on hepatic and muscle insulin sensitivity. Am J Physiol—Endocrinol Metab.

[41] Norum JH, Skarpen E, Brech A, Kuiper R, Waaler J, Krauss S (2018). The tankyrase inhibitor G007-LK inhibits small intestine LGR5 + stem cell proliferation without altering tissue morphology. Biol Res.

[42] Solberg NT, Waaler J, Lund K, Mygland L, Olsen PA, Krauss S (2018). TANKYRASE inhibition enhances the antiproliferative effect of PI3K and EGFR inhibition, mutually affecting β-CATENIN and AKT signaling in colorectal cancer. Mol Cancer Res.

[43] Li N, Wang Y, Neri S, Zhen Y, Fong LWR, Qiao Y (2019). Tankyrase disrupts metabolic homeostasis and promotes tumorigenesis by inhibiting LKB1-AMPK signalling. Nat Commun.

[44] Su Z, Deshpande V, James DE, Stöckli J (2018). Tankyrase modulates insulin sensitivity in skeletal muscle cells by regulating the stability of GLUT4 vesicle proteins. J Biol Chem.

[45] Nathubhai A, Haikarainen T, Koivunen J, Murthy S, Koumanov F, Lloyd MD (2017). Highly potent and isoform selective dual site binding tankyrase/wnt signaling inhibitors that increase cellular glucose uptake and have antiproliferative activity. J Med Chem.

[46] Malin SK, Kashyap SR, Hammel J, Miyazaki Y, DeFronzo RA, Kirwan JP (2014). Adjusting glucose-stimulated insulin secretion for adipose insulin resistance: an index of β-cell function in obese adults. Diabetes Care.

[47] Kim JY, Nasr A, Tfayli H, Bacha F, Michaliszyn SF, Arslanian S (2017). Increased lipolysis, diminished adipose tissue insulin sensitivity, and impaired β-cell function relative to adipose tissue insulin sensitivity in obese youth with impaired glucose tolerance. Diabetes.

[48] Donnelly KL (2005). Sources of fatty acids stored in liver and secreted via lipoproteins in patients with nonalcoholic fatty liver disease. J Clin Investig.

[49] Hussey S, Lum H, Alvarez A, Cipriani Y, Garduño-Garcia J, Anaya L (2014). A sustained increase in plasma NEFA upregulates the Toll-like receptor network in human muscle. Diabetologia.

[50] Samuel V, Shulman G (2012). Mechanisms for insulin resistance: common threads and missing links. Cell.

[51] Finelli C, Tarantino G (2013). What is the role of adiponectin in obesity related non-alcoholic fatty liver disease?. World J Gastroenterol.

[52] Lo KA, Ng PY, Kabiri Z, Virshup D, Sun L (2016). Wnt inhibition enhances browning of mouse primary white adipocytes. Adipocyte.

[53] Seale P, Conroe HM, Estall J, Kajimura S, Frontini A, Ishibashi J (2011). Prdm16 determines the thermogenic program of subcutaneous white adipose tissue in mice. J Clin Investig.

[54] Feldmann HM, Golozoubova V, Cannon B, Nedergaard J (2009). UCP1 ablation induces obesity and abolishes diet-induced thermogenesis in mice exempt from thermal stress by living at thermoneutrality. Cell Metab.

[55] Kopecky J, Clarke G, Enerbäck S, Spiegelman B, Kozak LP (1995). Expression of the mitochondrial uncoupling protein gene from the aP2 gene promoter prevents genetic obesity. J Clin Investig.

[56] Bai P, Cantó C, Oudart H, Brunyánszki A, Cen Y, Thomas C (2011). PARP-1 inhibition increases mitochondrial metabolism through SIRT1 activation. Cell Metab.

[57] Pirinen E, Cantó C, Jo Y, Morato L, Zhang H, Menzies K (2014). Pharmacological inhibition of poly(ADP-ribose) polymerases improves fitness and mitochondrial function in skeletal muscle. Cell Metab.

[58] Marra M, Scalfi L, Contaldo F, Pasanisi F (2004). Fasting respiratory quotient as a predictor of long-term weight changes in non-obese women. Ann Nutr Metab.

[59] Zurlo F, Lillioja S, Esposito-Del Puente A, Nyomba BL, Raz I, Saad MF (1990). Low ratio of fat to carbohydrate oxidation as predictor of weight gain: study of 24-h RQ. Am J Physiol.

[60] Liu C, Lin JD (2011). PGC-1 coactivators in the control of energy metabolism. Acta Biochim Biophys Sin.

[61] Busiello RA, Savarese S, Lombardi A (2015). Mitochondrial uncoupling proteins and energy metabolism. Front Physiol.

[62] Summermatter S, Shui G, Maag D, Santos G, Wenk MR, Handschin C (2013). PGC-1α improves glucose homeostasis in skeletal muscle in an activity-dependent manner. Diabetes.

