# Supplementary material for: Tankyrase inhibition ameliorates lipid disorder via suppression of PGC-1α PARylation in db/db mice
Source: Int J Obes (Lond). 2020 Apr 21;44(8):1691–702. doi: 10.1038/s41366-020-0573-z (PMC7381423; doi:10.1038/s41366-020-0573-z)
Supplement: Supplementary file 1 — Wang et al. Supplemental material [file 41366_2020_573_MOESM1_ESM.docx]

**Wang *et al*., Supplementary information**

**Table S1. Primers used in the study.**

| Target | Primer sequences | Reference |
| --- | --- | --- |
| *Srebp1a* | Forward 5´-CATGGACGAGCTGGCCTTCG-3´  Reverse 5´-GGAAGTCACTGTCTTGGTTGTTGA-3´ | This study |
| *Pparγ* | Forward 5´-GGAAGACCACTCGCATTCCTT-3´  Reverse 5´-GTAATCAGCAACCATTGGGTCA-3´ | (Yan *et al,* 2014) |
| *Pparα* | Forward 5´-CCGGCTCCGAACATTGGTGT -3´  Reverse 5´- AATGCACCACGCCGTGAGAA-3´ | This study |
| *Sdhb* | Forward 5´-TGACGTCAGGAGCCAAAAT -3´  Reverse 5´-GCTTGTGCCCCTCGACAG -3´ | This study |
| *Mdh2* | Forward 5´-CTCCTGCCAGTAGCTCCG -3´  Reverse 5´-TCCCAGGACAGCCACTTTAG -3´ | This study |
| *Ucp1* | Forward 5´- GCATTCAGAGGCAAATCAGC-3´  Reverse 5´- GCCACACCTCCAGTCATTAAG-3´ | (Garcia *et al*, 2016) |
| *Fat/Cd36* | Forward 5´-GGCTAAATGAGACTGGGACC-3  Reverse 5´-CATCACCACTCCAATCCCAAG-3´ | (Yeh *et al*, 2009) |
| *Aco1* | Forward 5´-ACCTTGCTTCACCAGGCCAC-3´  Reverse 5´-TGTCTGCGCATAAGTGCCCG-3´ | This study |
| *Cpt1β* | Forward 5´-TGATCATGTATCGCCGCAAACT-3´  Reverse 5´-CATCTGGTAGGAGCACATGG-3´ | (Yeh *et al*, 2009) |
| *Pgc-1α* | Forward 5´-CTGGGTGGATTGAAGTGGTG-3´  Reverse 5´-TCAGTGCATCAAATGAGGGC-3´ | (Yeh *et al*, 2009) |
| *Tnfα* | Forward 5´-CTTCTGTCTACTGAACTTCGGG -3´  Reverse 5´-CAGGCTTGTCACTCGAATTTTG -3´ | (Gabunia *et al*, 2016) |
| *Pck1* | Forward 5′-CGTGGCCGAGACTAGCGATC -3′  Reverse 5′-ATAATGGGGCACTGGCTGGC -3′ | This study |
| *G6pc* | Forward 5′- AACGCCCGTATTGGTGGGTC-3′  Reverse 5′-GGCATGGCCAGAGGGACTTC -3′ | This study |
| *18S rRNA* | Forward 5´-GAGGGACAAGTGGCGTTCAG-3´  Reverse 5´-ATCACGAATGGGGTTCAACG-3´ | This study |

**Table S2. Antibodies used in the study.**

| Antibody | Source | Manufacturer | Reference |
| --- | --- | --- | --- |
| TNKS 1/2 | Rabbit | Santa Cruz Biotechnology | sc-8337 |
| AXIN1 | Rabbit | Cell Signaling Technology | #3323S |
| phospho-HSL(Ser660) | Rabbit | Cell Signaling Technology | #4126S |
| ATGL | Rabbit | Cell Signaling Technology | #2138S |
| FAS | Rabbit | Abcam^®^ | ab128870 |
| phospho-ACC(Ser79) | Rabbit | Cell Signaling Technology | #11818S |
| UCP1 | Rabbit | Abcam^®^ | ab10983 |
| AdipoQ | Rabbit | Biorbyt | orb 137986 |
| Poly(ADP-ribose) | Rabbit | Enzo Life Sciences | ALX-210-890-R100 |
| PGC-1α | Rabbit | EMD Millipore | AB3242 |
| PGC-1α | Mouse | Calbiochem^®^ | ST1202 |
| VDAC/Porin | Rabbit | Abcam | ab15895 |
| OXPHOS | Mouse | Abcam | ab110413 |
| actin | Mouse | Sigma-Aldrich | A3853 |
| Ki-67 | Rabbit | Bethyl Laboratories | IHC-00375 |
| IRDye 800-rabbit IgG | Donkey | LI-COR | #926-32213 |
| IRDye 680-mouse IgG | Donkey | LI-COR | #926-68072 |
| AlexaFluor 594-rabbit IgG | Donkey | Invitrogen | A-21207 |

**Figure S1. G007-LK has no effect on intestinal epithelial cell proliferation in *db/db* and *db/+* mice.**

Representative immunofluorescent images of Ki-67 expression at the base of the intestinal crypts of G007-LK-treated and non-treated *db/db* (upper panels) and *db/+* (lower panels) mice (*n* = 4 per group). Intestinal sections were blocked with CAS-block (Invitrogen, Carlsbad, CA), incubated with rabbit anti-Ki-67 antibody in Dako REAL™ Antibody Diluent (Dako, Santa Clara, CA), followed by AlexaFluor 594 donkey anti-rabbit IgG and Hoechst (Sigma-Aldrich, St. Louis, MO, USA). Slides were mounted in Vectashield (Vector Laboratories, Burlingame, CA) and examined with Zeiss Axioplan2 microscope (Carl Zeiss Microscopy). Images were captured with an Andor Zyla sCMOS camera (Andor Technology Ltd, Belfast, Ireland). Scale bar, 100 µm.

**
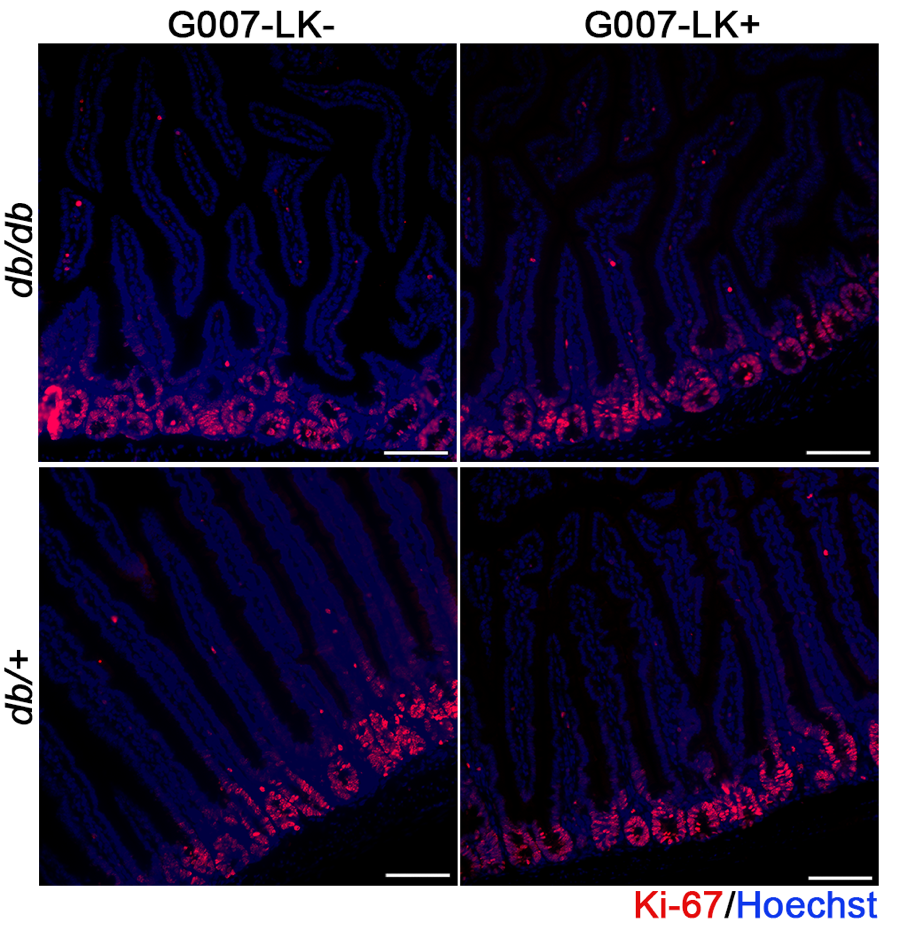
**

**Figure S2. G007-LK treatment leads to a trend of reduced adipocyte size in WAT.**

A Hematoxylin and eosin (HE) stained epididymal fat pads of control and G007-LK treated *db/db* mice. Frozen WAT sections (25 µm) were fixed with methanol and stained with HE. Slides were mounted in Eukitt hardening mounting medium (Sigma-Aldrich, St Louis, MO). Images were generated using 3DHISTECH Pannoramic 250 FLASH II digital slide scanner (Budapest, Hungary) at Genome Biology Unit supported by HiLIFE and the Faculty of Medicine, University of Helsinki, and Biocenter Finland.

B The violin plot showing the distribution of adipocyte size. The Image analysis was carried out by computer assisted segmentation with open- source Cell Profiler –software (Lamprecht *et al*, 2007). In the analysis *db/db* n = 4; *db/db* + G007-LK n = 6.

In B, the line represents the median of the data set and the dashed lines the upper and lower quartiles.


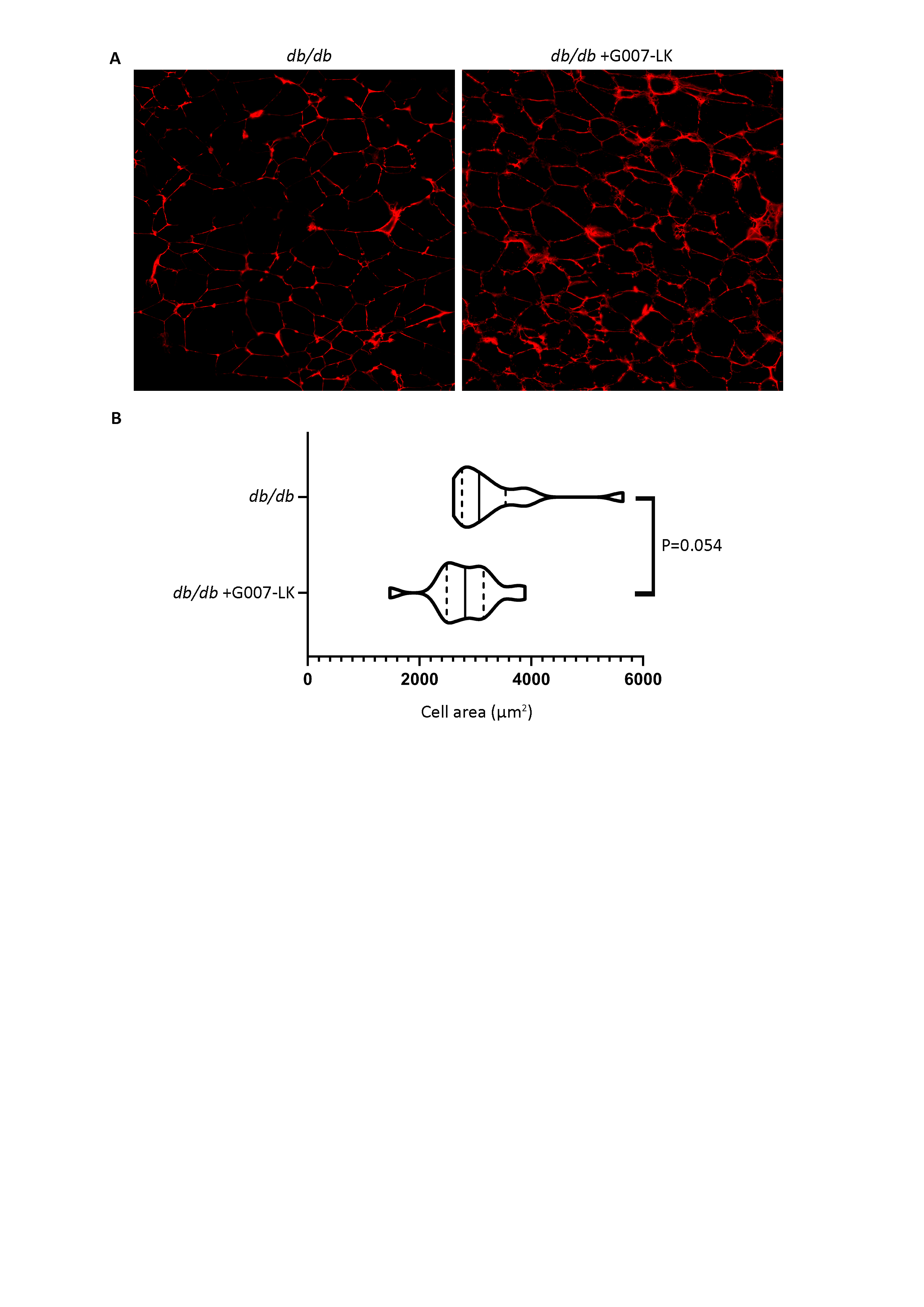


**Figure S3. TNKS inhibitor treatment has no effect on physiological parameters or insulin sensitivity.**

A-D Average food consumption per day (A); Voluntary wheel-running activity over 72 consecutive hours at 12 weeks of treatment (B, C) was measured after a two-day habituation to a wireless running wheel (ENV-044 model; Med Associates, Inc., St. Albans, Vermont, USA). Body temperature at 9 weeks of treatment (D) was measured with a rectal thermometer (DM 852, Ellab Instruments, Rodovre, Denmark).

E, F Fasting blood glucose level was measured at 0, 4, 8 and 15 weeks of treatment (E). Shortly, for glucose and insulin measurements, blood was obtained from the saphenous vein of mice fasted for 6 h. Blood glucose was measured using a Contour® Glucometer (Bayer, Elkhart, IN, USA). Fasting serum insulin level was measured at 0, 4, and 8 weeks of treatment (F). Serum insulin was determined with an Ultra-Sensitive Mouse Insulin ELISA Kit (Crystal Chem, Downers Grove, IL, USA).

G, H Intraperitoneal insulin tolerance (ITT) test was performed at 11 weeks of treatment. For ITT fasted mice were injected intraperitoneally with 0.4 U/kg insulin followed by measuring blood glucose every 30 min up to 120 min.

Error bars represent ± SEM. In (A-H), *db/db* G007-LK- *n* = 6; *db/db* G007-LK+ *n* = 10; *db/+* G007-LK- *n* = 9; *db/+* G007-LK+ *n* = 10.

**
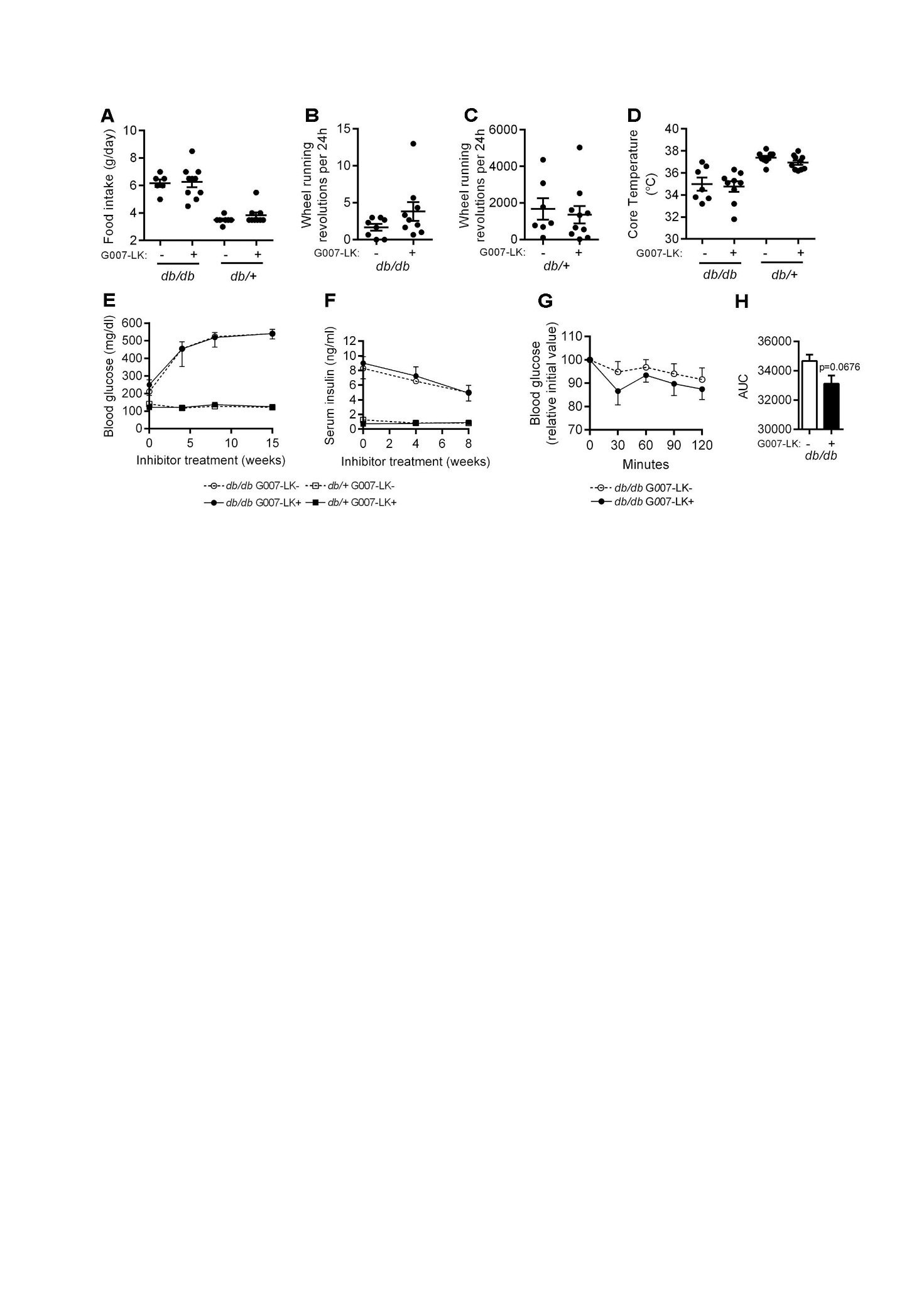
**

**Figure S4. G007-LK inhibits TNKS activity in WAT and muscle of *db/db* mice.**

A, B Representative immunoblot of TNKS1/2 (A) and AXIN1 (B) in WAT of G007-LK-treated and non-treated mice. In (A), *db/db* G007-LK- *n* = 6; *db/db* G007-LK+ *n* = 8; *db/+* G007-LK- and *db/+* G007-LK+ *n* = 7. In (B), *db/db* G007-LK- *n* = 3; *db/db* G007-LK+ *n* = 5; *db/+* G007-LK- and *db/+* G007-LK+ *n* = 7.

C, D Representative immunoblot of TNKS1/2 (C) and AXIN1 (D) in muscle of G007-LK-treated and non-treated mice. In (C), *n* = 4 in each group. In (D), *db/db* G007-LK- *n* = 3; *db/db* G007-LK+, *db/+* G007-LK- and *db/+* G007-LK+ *n* = 4.

The graphs represent quantifications of the indicated proteins normalized to actin. Error bars represent ± SEM, two-tailed *t* test. * *p* < 0.05, ** *p* < 0.01.


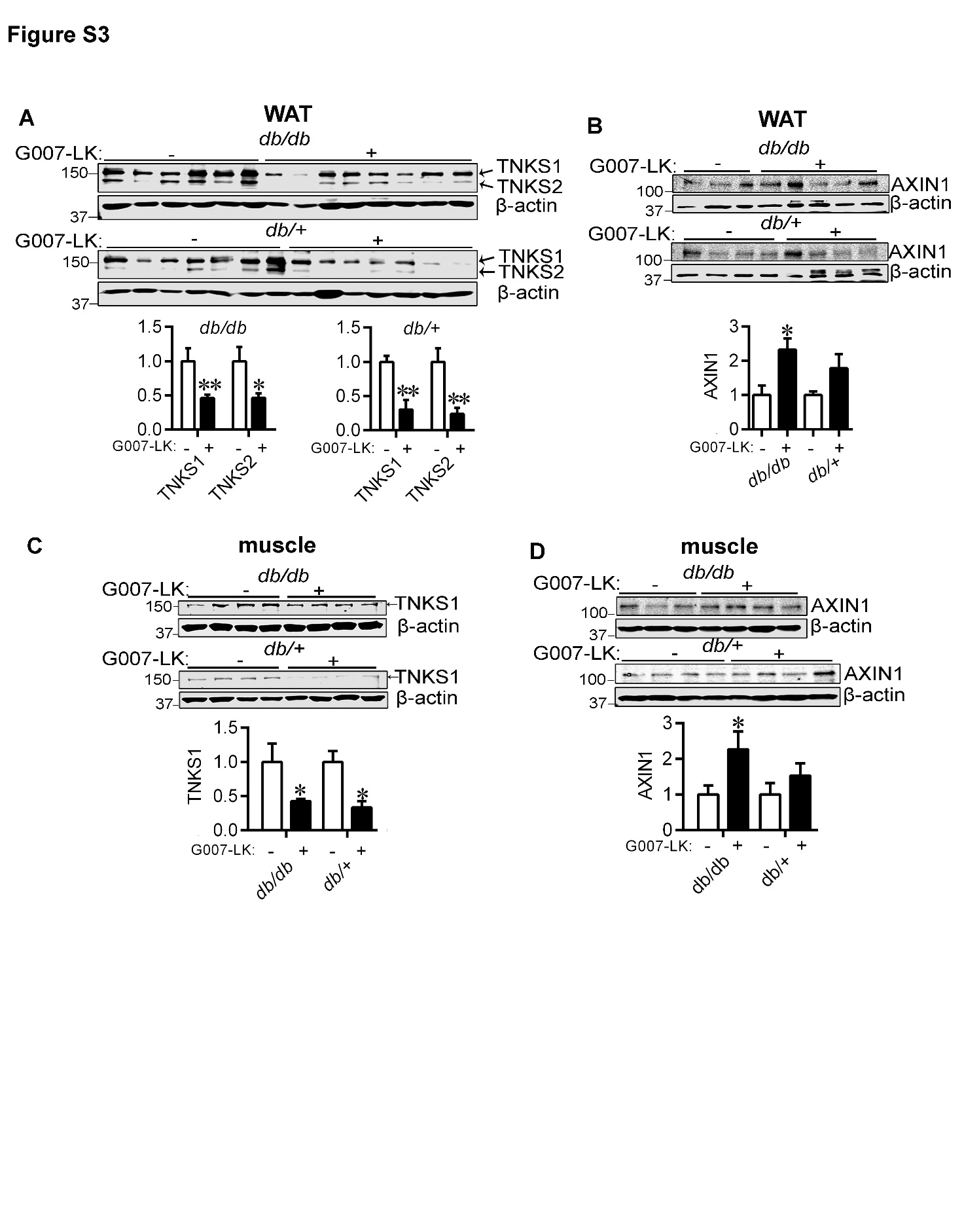


**Figure S5. G007-LK has no effect on molecules associated with lipid metabolism in WAT in db/+ mice.**

A, B Representative immunoblot (A) and quantification (B) of ATGL and phospho-HSL in WAT of G007-LK-treated and control db/+ mice.

C-F Representative immunoblots (C, E) and corresponding quantifications (D, F) of adiponectin (AdipQ) in WAT (C, D) and serum (E, F) of G007-LK-treated and control mice after 15 weeks of treatment.

The graphs represent quantifications of the indicated proteins normalized to actin (B, D) or quantification of the individual bands on the blots (F). Error bars represent ± SEM.

**
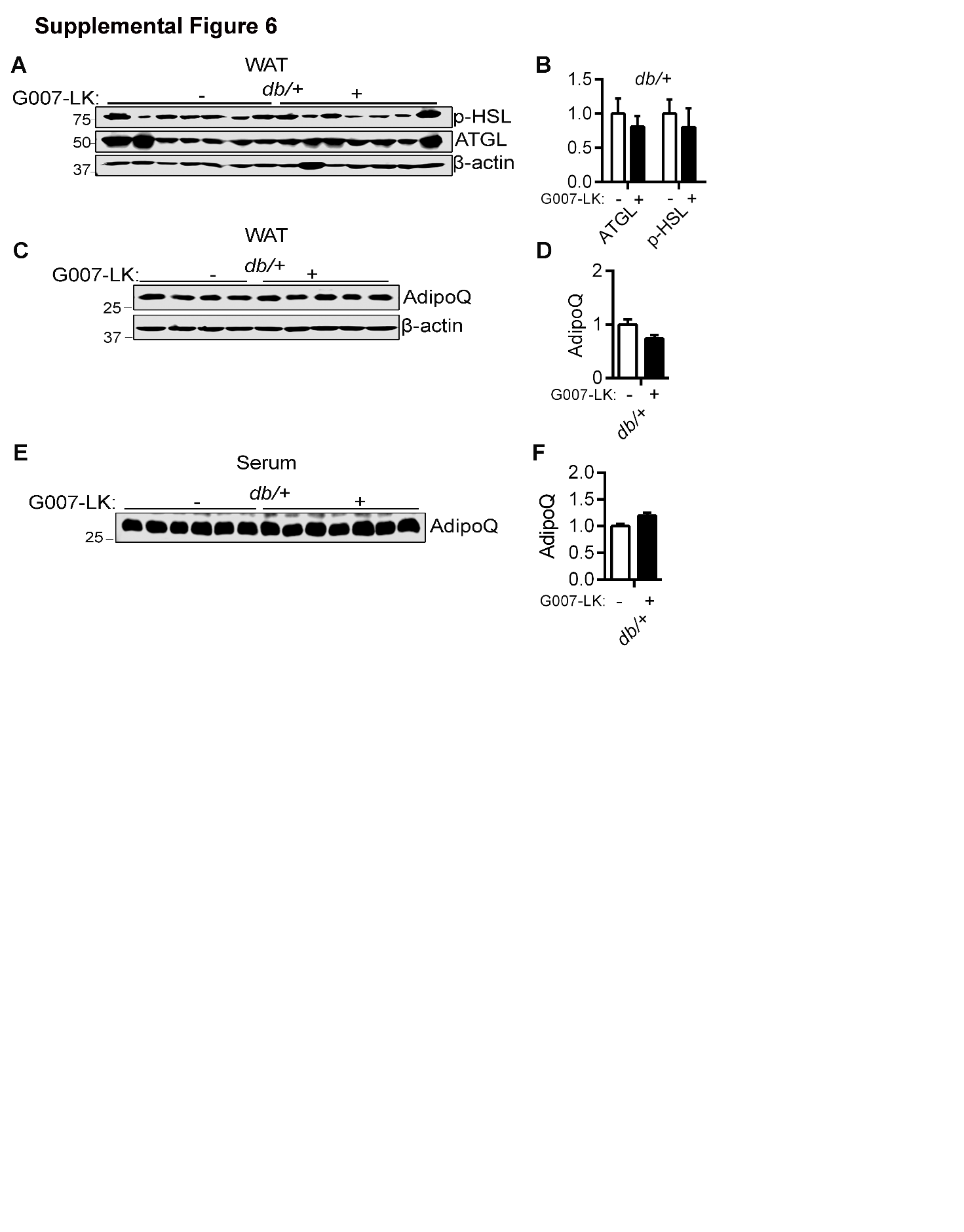
**

**Figure S6. Inhibition of TNKSs has no significant effect on lipogenesis in WAT of *db/db* and *db/+* mice.**

A G007-LK treatment for 15 weeks did not affect FAS or inactive p-ACC levels in WAT in either *db/db* or *db/+* mice. Quantifications of the blots normalized to actin are shown in the graphs. Immunoblots are representative of three separate experiments (for *db/db* mice, n = 4 for non-treated and n = 5 for G007-LK-treated; for *db/+* mice, n = 4 for non-treated and G007-LK-treated).

B Quantitative RT-PCR analyses of WAT of G007-LK-treated vs. non-treated mice showed comparable expression levels of transcriptional regulators of lipogenesis and adipogenesis, *Srebp1a* and *Pparγ,* and Fat/*Cd36,* a target gene of *Pparγ*. Error bars represent ± SEM.

**
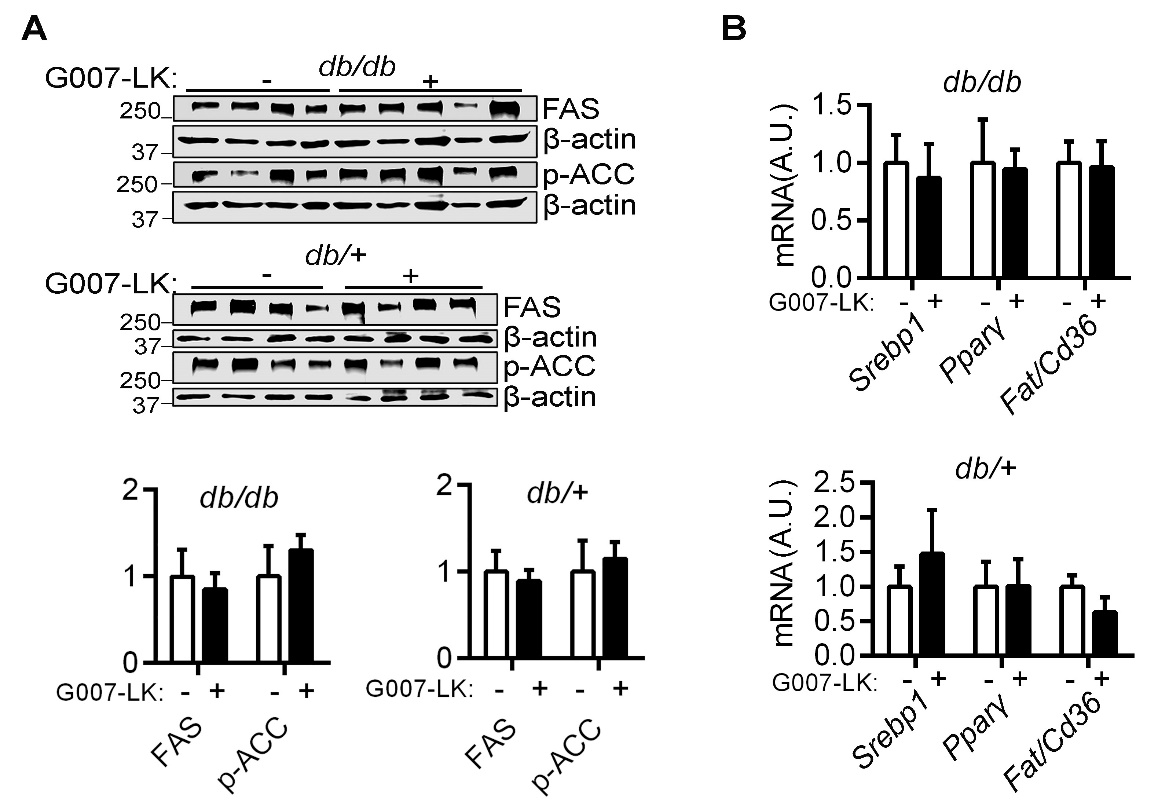
**

**Figure S7. G007-LK has no effect on TNKS activity genes associated with lipid utilisation or gluconeogenesis in liver of *db/db* and *db/+* mice.**

A Representative immunoblot of TNKS1 and TNKS2 in liver of *db/db* and *db/+* mice (*db/db* mice, n = 4 per group; *db/+* mice, n = 4 for nontreated and n = 5 for G007-LK-treated). G007-LK reduces the expression of TNKS1 and TNKS2 in liver in *db/+* mice. *db/db* mice show a trend of reduced expression of TNKS1 and TNKS2.

B Representative immunoblot showing that G007-LK has no significant effect on AXIN1 level in liver of *db/db* or *db/+* mice (*db/db* mice, n = 4 for nontreated and n = 3 for G007-LK-treated; *db/+* mice, n = 3 for nontreated and n = 5 for G007-LK-treated).

C Quantitative RT-PCR analyses show comparable expression levels of transcriptional regulators of lipid metabolism (*Srebp1a*, *Pparα*, *Pgc-1α*) and genes responsible for fatty acid oxidation (*Aco1*, *Cpt1α*) and lipid uptake (*Fat/Cd36*) and gluconeogenesis (*Pck1*, *G6pc*) in liver of G007-LK-treated and non-treated mice.

D Representative immunoblot, which reveals no difference in the expression level of PGC-1α in liver of G007-LK-treated and non-treated *db/db* mice (n = 4 for nontreated and n = 5 for G007-LK-treated). Immunoblots are representative of three separate experiments. The graphs represent quantifications of the indicated proteins normalized to actin. Error bars represent ± SEM.
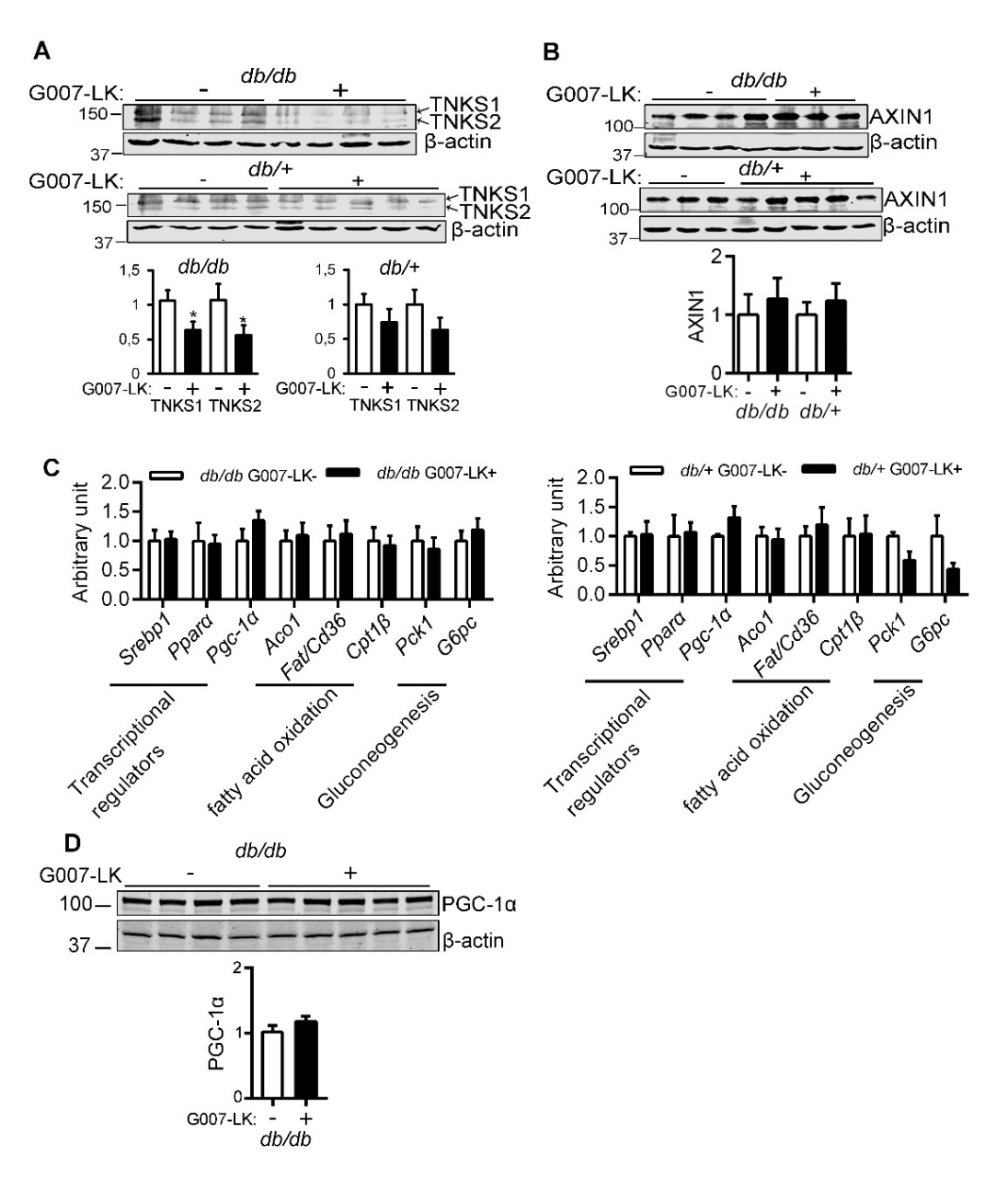


**Figure S8. G007-LK has no effect on genes associated with fatty acid uptake and oxidation in muscle of db/+ mice.**

A qRT-PCR analyses of genes associated with fatty acid oxidation in muscle of G007-LK-treated and control *db/+* mice.

B C Representative immunoblot (B) and quantification (C) of PGC-1α in muscle of G007-LK-treated and control *db/+* mice. Error bars represent ± SEM.

**
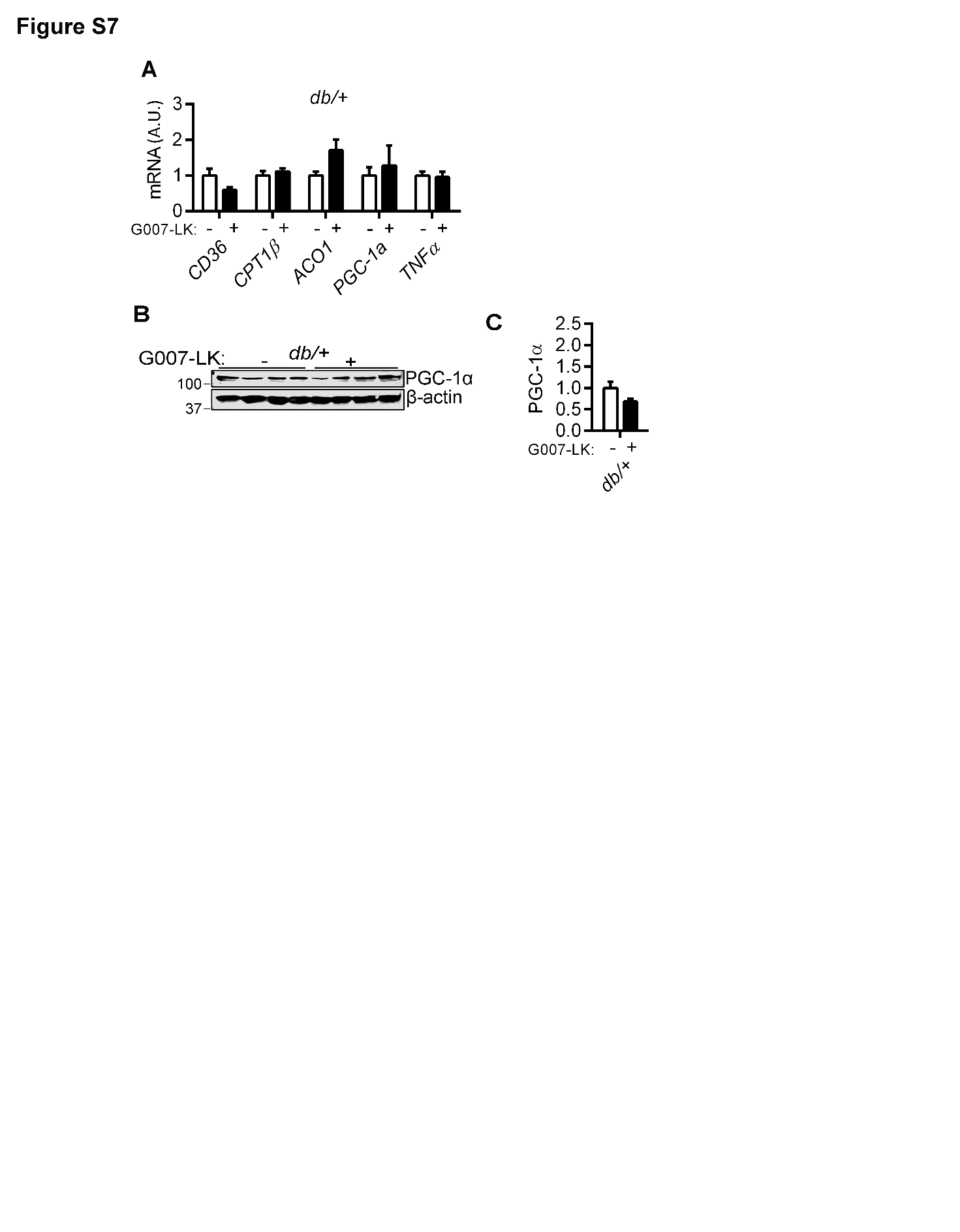
**

**References**

Gabunia K, Ellison S, Kelemen S, Kako F, Cornwell WD, Rogers TJ, Datta PK, Ouimet M, Moore KJ, Autieri MV (2016) IL-19 Halts Progression of Atherosclerotic Plaque, Polarizes, and Increases Cholesterol Uptake and Efflux in Macrophages*. Am J Pathol* 186: 1361-1374.

Garcia RA, Roemmich JN, Claycombe KJ (2016) Evaluation of markers of beige adipocytes in white adipose tissue of the mouse*. Nutr Metab (Lond)* 13: 24-016-0081-2.

Yan Z, Zhang H, Maher C, Arteaga-Solis E, Champagne FA, Wu L, McDonald JD, Yan B, Schwartz GJ, Miller RL (2014) Prenatal polycyclic aromatic hydrocarbon, adiposity, peroxisome proliferator-activated receptor (PPAR) gamma methylation in offspring, grand-offspring mice*. PLoS One* 9: e110706.

Yeh TY, Beiswenger KK, Li P, Bolin KE, Lee RM, Tsao TS, Murphy AN, Hevener AL, Chi NW (2009) Hypermetabolism, hyperphagia, and reduced adiposity in tankyrase-deficient mice*. Diabetes* 58: 2476-2485.

Lamprecht MR, Sabatini DM, Carpenter AE (2007) CellProfiler: free, versatile software for automated biological image analysis. Biotechniques 42(1):71-75
